# Sustained Activation of Akt Elicits Mitochondrial Dysfunction to Block *Plasmodium falciparum* Infection in the Mosquito Host

**DOI:** 10.1371/journal.ppat.1003180

**Published:** 2013-02-28

**Authors:** Shirley Luckhart, Cecilia Giulivi, Anna L. Drexler, Yevgeniya Antonova-Koch, Danielle Sakaguchi, Eleonora Napoli, Sarah Wong, Mark S. Price, Richard Eigenheer, Brett S. Phinney, Nazzy Pakpour, Jose E. Pietri, Kong Cheung, Martha Georgis, Michael Riehle

**Affiliations:** 1 Department of Medical Microbiology and Immunology, School of Medicine, University of California, Davis, Davis, California, United States of America; 2 Department of Molecular Biosciences, School of Veterinary Medicine, University of California, Davis, Davis, California, United States of America; 3 Medical Investigations of Neurodevelopmental Disorders (MIND) Institute, University of California, Davis, Davis, California, United States of America; 4 Department of Entomology, University of Arizona, Tucson, Arizona, United States of America; 5 Genome and Biomedical Sciences Center, University of California, Davis, Davis, California, United States of America; Stanford University, United States of America

## Abstract

The overexpression of activated, myristoylated Akt in the midgut of female transgenic *Anopheles stephensi* results in resistance to infection with the human malaria parasite *Plasmodium falciparum* but also decreased lifespan. In the present study, the understanding of mitochondria-dependent midgut homeostasis has been expanded to explain this apparent paradox in an insect of major medical importance. Given that Akt signaling is essential for cell growth and survival, we hypothesized that sustained Akt activation in the mosquito midgut would alter the balance of critical pathways that control mitochondrial dynamics to enhance parasite killing at some cost to survivorship. Toxic reactive oxygen and nitrogen species (RNOS) rise to high levels in the midgut after blood feeding, due to a combination of high NO production and a decline in FOXO-dependent antioxidants. Despite an apparent increase in mitochondrial biogenesis in young females (3 d), energy deficiencies were apparent as decreased oxidative phosphorylation and increased [AMP]/[ATP] ratios. In addition, mitochondrial mass was lower and accompanied by the presence of stalled autophagosomes in the posterior midgut, a critical site for blood digestion and stem cell-mediated epithelial maintenance and repair, and by functional degradation of the epithelial barrier. By 18 d, the age at which *An. stephensi* would transmit *P. falciparum* to human hosts, mitochondrial dysfunction coupled to Akt-mediated repression of autophagy/mitophagy was more evident and midgut epithelial structure was markedly compromised. Inhibition of RNOS by co-feeding of the nitric-oxide synthase inhibitor *L*-NAME at infection abrogated Akt-dependent killing of *P. falciparum* that begins within 18 h of infection in 3–5 d old mosquitoes. Hence, Akt-induced changes in mitochondrial dynamics perturb midgut homeostasis to enhance parasite resistance and decrease mosquito infective lifespan. Further, quality control of mitochondrial function in the midgut is necessary for the maintenance of midgut health as reflected in energy homeostasis and tissue repair and renewal.

## Author Summary

Malaria is a major public health problem in the world and various strategies are under development for control, including vaccines and transgenic mosquitoes that block parasite transmission. We previously reported that overexpression of the major signaling protein Akt in the midgut of female *Anopheles stephensi* mosquitoes could impart resistance to infection with the most important human malaria parasite and also reduce the duration of mosquito infectivity to human hosts. However, to use this strategy for malaria transmission control in endemic areas, we must understand the mechanism by which parasites are killed to ensure that transmission of other human pathogens (e.g., viruses, nematodes) is not unexpectedly enhanced and to allow the design of rational, preventive interventions. Here, we report that overexpression of a constitutively active Akt in the mosquito midgut alters important cellular, and in particular, mitochondrial processes – in a manner similar to Akt control of these processes in mammalian cells – to generate high levels of toxic compounds that kill parasites within hours after infection. However, the same alterations in mitochondrial processes that result in parasite killing ultimately reduce mosquito infective lifespan for transmission, indicating that mitochondrial dynamics in the mosquito midgut could be targeted for multi-faceted genetic control of mosquito biology to reduce malaria transmission.

## Introduction

Malaria is one of the greatest public health threats worldwide and is caused by infection with protozoan parasites of the genus *Plasmodium* that are transmitted by *Anopheles* mosquitoes. Shortly after an infective bloodmeal is consumed by the female mosquito, which can occur as early as 3 d of age, zygotes form and develop into motile ookinetes in the midgut lumen. Ookinetes must successfully traverse the midgut epithelium to form non-motile oocysts that grow and develop on the outside of the midgut for a minimum of 12 d. Within 14–16 d of ingesting a parasite-containing blood meal (or at 17–19 d post-emergence of the mosquito), oocyst-derived sporozoites invade the salivary glands to yield a mosquito that is infective to humans for the duration of her life. Despite this need for lengthy development, only a small percentage of mosquitoes under natural conditions live long enough to become fully infective [1]–[3].

Akt is a key signaling molecule in nearly all eukaryotes and regulates a variety of physiological processes in a tissue dependent manner. In mosquitoes, Akt regulates immunity, lifespan, reproduction, metabolism and diapause [4]. We previously demonstrated that increased Akt signaling in the midgut of the female malaria mosquito *Anopheles stephensi* disrupted development of the human malaria parasite *Plasmodium falciparum* and concurrently reduced the duration that mosquitoes are infective to humans [5]. Specifically, overexpression of constitutively active Akt (myristoylated Akt or myrAkt) in heterozygous (HT) transgenic *An. stephensi* reduced parasite infection by 60–99% relative to non-transgenic (NTG) controls. Of those mosquitoes that were infected, we observed a 75–99% reduction in parasite load. Homozygous (HM) transgenic mosquitoes were resistant to parasite infection. The increase in midgut-specific Akt signaling also reduced the average mosquito lifespan by 18–20% and the window of opportunity to transmit malaria parasites by 50% relative to controls. Thus, activation of Akt signaling reduced the number of infected mosquitoes, the number of malaria parasites per infected mosquito, and the duration of mosquito infectivity relative to NTG controls. While these findings are significant, the safe application of this or any similar strategy for malaria transmission control requires identification of the mechanism(s) whereby parasites are killed to ensure that transmission of other human pathogens (e.g., viruses, nematodes) is not unexpectedly enhanced and to allow the design of rational, preventive interventions.

The signaling protein Akt is not only a key mediator in insulin and insulin-like growth factor signaling (IIS), but it also integrates signals from other growth factor-activated tyrosine kinase receptors as well as activated G-protein-coupled receptors and integrins to regulate a wide array of downstream proteins involved in cell growth, cell survival, and metabolism [6]. Although activation of Akt is required for fundamental processes, sustained activation of the Akt pathway – as observed in PTEN haploinsufficient or null mice – can lead to abnormal behavior [7], [8], disrupted autophagy [9], and mitochondrial dysfunction with accumulation of mtDNA deletions [8]. Furthermore, enhanced or defective clearance of damaged mitochondria by autophagy or mitophagy [10], [11] has been reported in several disorders with dysregulation of Akt [12], [13], [14]. Increases in PTEN, in contrast, lead to increased oxidative phosphorylation and increased tumor resistance [15].

Removal of damaged mitochondria occurs via general autophagy or by a selective form of autophagy termed mitophagy [16]. In general, signals that *promote* mitophagy and autophagy, in addition to oxidative stress-mediated damage, include activation of the stress-associated kinases (p38 MAPK and JNK), ERK-dependent signaling, energy deprivation, some pro-inflammatory cytokines as well as Toll-like receptor (TLR) and peptidoglycan recognition protein (PGRP) signaling [17], [18]. Signals that *inhibit* mitophagy and autophagy include the signaling axis surrounding Akt (phosphatidylinositol-3 kinase [PI-3K] upstream of Akt and target of rapamycin [TOR] downstream of Akt), allergic inflammation-associated signals including interleukin (IL)-4, IL-13, and some microbial virulence factors [17], [18]. Autophagy is critical for the regulation and coordination of cellular homeostasis, epithelial barrier integrity, stem cell maintenance and differentiation, lifespan, and immunity [17]–[21]. For example, autophagy in *C. elegans daf-2* mutants – which are both long-lived and infection-resistant – is necessary and sufficient for pathogen resistance and lifespan extension under reduced IIS activation [22], [23]. Thus, balance between mitochondrial biogenesis and these positive and negative signals for the clearance of damaged mitochondria is required for adequate levels of steady-state functional mitochondria.

In light of observations from nematodes to mammals and the phenotypes of enhanced anti-parasite resistance and reduced lifespan in our transgenic mosquitoes, we hypothesized that myrAkt overexpression in the midgut of *An. stephensi* would lead to mitochondrial dysfunction that could impact resistance to *P. falciparum* infection and infective lifespan. Specifically, we predicted that Akt overexpression would increase steady-state oxidative stress through a reduction in antioxidants, including FOXO-dependent mitochondrial MnSOD and perhaps glutathione-*S*-transferases [24], [25] and through enhanced NO production, based on previous observations of ROS-induced *NOS* expression in *An. stephensi* cells [26]. Given the reciprocal interaction between PTEN levels and mitochondrial dysfunction [8], [15], we hypothesized that reduced oxidative phosphorylation, resulting from oxidative damage by increased toxic reactive nitrogen and oxygen species (RNOS) contributed in part perhaps from enhanced mitochondrial ROS production, would feedback to increase damage if not accompanied by concomitant increases in antioxidant and repair enzymes. While a significant upregulation of toxic RNOS could be sufficient to kill parasites, we reasoned that RNOS-damaged midgut architecture could also contribute to anti-parasite resistance and, at the same time, alter infective lifespan. For example, low MnSOD activity, which in the presence of normal or high NO production would result in peroxynitrite formation within mitochondria [27], [28], could disrupt the activity of several targets, in particular, Complexes I [9], [29] and V [30], [31] and, possibly, remaining MnSOD [32], [33]. Although the resulting high oxidative stress should be a potent signal for activating autophagy and/or mitophagy, Akt overexpression would antagonize RNOS-mediated activation, resulting in reduced clearance of damaged mitochondria, altered biogenesis, and concomitantly higher RNOS. Hence, despite the reduction in parasite infection, Akt-induced metabolic changes would lead to significantly altered mitochondrial dynamics and damage to the midgut epithelium, the primary site for nutrient acquisition from ingested blood.

Previous studies from Oliveira et al. [34] and Kumar et al. [35] using a strain of *Anopheles gambiae* (L3–5) genetically selected for refractoriness (R) to infection with the simian parasite *Plasmodium cynomolgi*, reported that resistance to infection was related to a heightened state of oxidative stress resulting from lower antioxidant defenses based on morphological and microarray mRNA expression analysis. In particular, they observed decreased State 3-dependent oxygen uptake in midgut and thorax, increased midgut ROS production (from Nox, Duox or mitochondria), and decreased fat stores with increased transcript expression of several glycolytic enzymes. When Oliveira et al. [34] silenced the mitochondrial adenine nucleotide transporter (ANT) in sensitive mosquitoes, they observed increased ROS production and a recapitulation of “resistance to infection” observed in R mosquitoes. Although the only glycolytic transcript increased was lactate dehydrogenase (LDH), the authors concluded that a “metabolic shift” sustained oxidative stress to favor killing of parasites, driving resistance to infection. A decrease in State 3-mediated oxygen uptake (especially in thorax and less in midgut) and increased LDH transcript, however, would not favor beta-oxidation of fatty acids, a mitochondrial process, to explain the decrease in fat deposits. Rather, these changes would favor glycolysis as a main source of ATP in an attempt to cope with the lower output of mitochondrial ATP. Midgut epithelium is a highly aerobic tissue, for which the electrolyte balance would not be supported by glycolysis alone. Thus, these changes do not appear to be “adaptive” with a temporary implication, rather they are terminal, dramatic changes that would result in increased resistance (less infection) but with a higher cost (shorter lifespan). Although this model of malaria resistance is appealing, it is not possible to definitively identify which (or any) of the detected metabolic changes define the mechanism of resistance given that resistance could result from altered expression of more than one gene in more than one tissue. Indeed, Oliveira et al. [34] suggested that the changes associated with increased resistance might be affected by a regulatory protein such as “a constitutively active transcription factor or a non-functional suppressor of a signal transduction pathway”. In this study, we have used a well-defined model of malaria resistance based on expression of constitutively active myrAkt in the midgut of female mosquitoes [5] to elucidate the mechanism underlying resistance to infection. Here, sustained activation of Akt functions broadly to control mitochondrial dynamics in malaria resistance. This control is initiated as an overproduction of NO and resistance is sustained as an imbalance of mitochondrial biogenesis and autophagy. This fundamental imbalance perturbs midgut homeostasis or “midgut health” to mediate the mosquito response to infection and the infective lifespan.

## Results

### myrAkt overexpression was associated with increased mitochondrial and cytoskeletal proteins and repression of MAPK signaling in *An. stephensi*

To identify the molecular targets affected by Akt overexpression in *An. stephensi*, we used differential LC-MS/MS to identify proteins that were over- or underrepresented in 3–5 d old, female myrAkt HM and HT *An. stephensi* relative to age-matched, NTG female *An. stephensi*. Analysis of the *An. gambiae* and *An. stephensi* genome data sets yielded largely concordant results; additional related matches from other mosquito species were detected through limited analyses of available data.

Among 477 identified proteins, a total of 38 proteins (excluding the transgenesis eye marker DsRed and multiple hits to the same protein in different mosquito genomes) were shared between HM and HT *An. stephensi* that were not evident or were reduced in NTG mosquitoes (**Table 1**). Notably, of these 38 proteins, a large proportion (14/38 or 37%) were associated with mitochondrial processes, including Krebs' cycle, electron transport chain subunit assembly, protein folding, and mitochondria-specific oxidative stress responses. The latter included an antioxidant enzyme, a peroxiredoxin V (PrxV) ortholog, and three additional proteins generally associated with oxidative stress response, including chaperones (heat shock protein), protein refolding (protein disulfide isomerase) and reduction of oxidative modifications (aldo-keto reductase). In *Drosophila melanogaster*, *prxV* ^−/−^ mutants exhibited reduced survivorship under oxidative stress, while overexpression of PrxV enhanced oxidative stress resistance and lifespan [36]. In other studies, PrxV was associated with redox regulation during bacterial infection of the midgut in *D. melanogaster*, but overexpression of PrxV was unexpectedly associated with reduced fly survivorship relative to controls following infection [37]. While the association of PrxV overexpression with reduced lifespan under some circumstances is intriguing, it is more likely that overexpressed PrxV here is a limited response to oxidative stress, perhaps protecting only a small subset of mitochondrial proteins [38].

**Table 1:** Proteins with over- or underrepresented peptide representation in myrAkt *An. stephensi* relative to NTG mosquitoes (mitochondrial proteins in bold).

Pathway or Process	Accession Number^1^	Protein Name	*P value*	Uniprot Number
**Over-represented proteins in myrAkt ** ***An. stephensi***				
Glycolysis	gi|55233926|gb|EAA01768.2|	phosphoglycerate mutase AGAP001420-PA [*Anopheles gambiae* str. PEST]	0.00081	Q7PXI5
**Krebs cycle**	**gi|55234034|gb|EAA01194.2|**	**succinyl-CoA synthetase small subunit AGAP001312-PA [** ***Anopheles gambiae*** ** str. PEST]**	**0.02400**	**Q7PXA8**
	**gi|157012490|gb|EAA01572.4|**	**mitochondrial malate dehydrogenase AGAP001903-PA [** ***Anopheles gambiae*** ** str. PEST]**	**0.00043**	**Q7PYE7**
	**gi|312371315|gb|EFR19537.1|**	**isocitrate dehydrogenase hypothetical protein AND_22267 [** ***Anopheles darlingi*** **]**	**0.00220**	**E3XEV8**
	**gi|157015178|gb|EAA12184.4|**	**aconitase AGAP007852-PA [** ***Anopheles gambiae*** ** str. PEST]**	**0.03200**	**Q7Q3F6**
**Complex II**	**gi|157014465|gb|EAA13601.3|**	**electron transfer flavoprotein subunit alpha AGAP004031-PA [** ***Anopheles gambiae*** ** str. PEST]**	**0.00008**	**Q7Q254**
	**gi|108879274|gb|EAT43499.1|**	**electron transfer flavoprotein beta-subunit [** ***Aedes aegypti*** **]**	**0.00036**	**Q17B68**
**Complex III**	**gi|157015986|gb|EAA11280.3|**	**Ubiquinol-cytochrome-c reductase complex core protein 2 AGAP006099-PA [** ***Anopheles gambiae*** ** str. PEST]**	**0.00410**	**Q7Q609**
**Complex IV**	**gi|55239920|gb|EAA10211.2|**	**cytochrome c oxidase polypeptide Vb AGAP008724-PA [** ***Anopheles gambiae*** ** str. PEST]**	**0.02300**	**Q7Q8A5**
**Complex V**	**gi|157012975|gb|EAA01034.4|**	**ATP synthase B chain**	**0.00000**	**Q7PWZ7**
	**gi|312382542|gb|EFR27965.1|**	**ATP synthase D chain hypothetical protein AND_04739 [** ***Anopheles darlingi*** **]**	**0.00350**	**E3WQT0**
	**gi|116128722|gb|EAA08884.3|**	**ATP synthase delta chain**	**0.06300**	**Q7QAP1**
	**gi|167873641|gb|EDS37024.1|**	**ATP synthase delta chain, mitochondrial [** ***Culex quinquefasciatus*** **]**	**0.00066**	**B0WYE7**
	**gi|208657613|gb|ACI30103.1|**	**mitochondrial F1F0-ATP synthase subunit coupling factor 6 [** ***Anopheles darlingi*** **]**	**0.07500**	**B6DDV7**
	**gi|116131730|gb|EAA05107.3|**	**ATP synthase D chain AGAP011131-PA [** ***Anopheles gambiae*** ** str. PEST]**	**0.01200**	**Q7QHC8**
	**gi|108882048|gb|EAT46273.1|**	**ATP synthase delta chain, mitochondrial [** ***Aedes aegypti*** **]**	**0.00059**	**Q17I02**
	**gi|312374969|gb|EFR22427.1|**	**ATP synthase superfamily by CDD hypothetical protein AND_15298 [** ***Anopheles darlingi*** **]**	**0.02200**	**E3X6N3**
Amino acid metabolism	gi|157012502|gb|EAA01733.3|	aspartate ammonia lyase AGAP001884-PA [*Anopheles gambiae* str. PEST]	0.00003	Q7PYD5
	gi|157013490|gb|EAL38923.3|	fumarylacetoacetate hydrolase AGAP011634-PA [*Anopheles gambiae* str. PEST]	0.04000	Q5TMH4
	gi|55235302|gb|EAA14864.2|	glutamine synthetase AGAP008988-PA [*Anopheles gambiae* str. PEST]	0.00190	Q7PWF1
Cytoskeleton/cell structure	gi|157018220|gb|EDO64247.1|	troponin t - AGAP002350-PE [*Anopheles gambiae* str. PEST]	0.00230	Q7PGE9
	gi|21299655|gb|EAA11800.1|	spectrin -AGAP006686-PA [*Anopheles gambiae* str. PEST]	0.00260	Q7Q515
	gi|157012939|gb|EAA01819.4|	microtubule-associated protein 1A or futsch AGAP001194-PA [*Anopheles gambiae* str. PEST]	0.00046	Q7PX34
	gi|157015210|gb|EAA12330.5|	myosin light chain AGAP007806-PA [*Anopheles gambiae* str. PEST]	0.03500	Q7PNE3
Protein synthesis	gi|55236286|gb|EAA13967.2|	60S ribosomal protein L12AGAP010065-PA [*Anopheles gambiae* str. PEST]	0.00100	Q7Q0Y7
	gi|114864969|gb|ABI83789.1|	40S ribosomal protein S28 [*Anopheles funestus*]	0.00490	Q06DF8
	gi|157015257|gb|EAA12468.5|	acidic ribosomal protein P1 AGAP007740-PA [*Anopheles gambiae* str. PEST]	0.00400	Q7PNA9
Oxidative stress response	**gi|157012845|gb|EAU75715.2|**	**Mitochondrial peroxiredoxin 5 AGAP001325-PA [** ***Anopheles gambiae*** ** str. PEST]**	**0.00140**	A0NH65
	gi|157019735|gb|EAL41666.3|	putative heat shock protein AGAP000941-PA [*Anopheles gambiae* str. PEST]	0.04000	F5HJ02
	**gi|157018744|gb|EAA06299.5|**	**Mitochondrial peptidyl-prolyl cis-trans isomerase AGAP000462-PA [** ***Anopheles gambiae*** ** str. PEST]**	**0.00170**	**Q7PS16**
	gi|157020742|gb|EAA03854.4|	aldo-keto reductase-like AGAP011053-PA [*Anopheles gambiae* str. PEST]	0.00000	Q5TX68
	gi|157013017|gb|EAL38666.3|	protein disulfide isomerase AGAP012407-PA [*Anopheles gambiae* str. PEST]	0.01100	Q5TMX9
	**gi|55236687|gb|EAA13612.2|**	**60 kD heat shock protein AGAP004002-PA [** ***Anopheles gambiae*** ** str. PEST]**	**0.00080**	**Q7Q270**
Electrolyte balance	gi|157017901|gb|EAA44868.4|	Na+/K+ ATPase alpha subunit AGAP002858-PA [*Anopheles gambiae* str. PEST]	0.00000	Q7PGN1
Salivary gland	gi|27372895|gb|AAO06821.1|	salivary antigen-5 related protein [*Anopheles stephensi*]	0.06800	Q8I6R0
	gi|27372911|gb|AAO06829.1|	salivary apyrase [*Anopheles stephensi*]	0.00340	Q8I6Q2
Others	gi|312377334|gb|EFR24188.1|	multiple Ig-domain containing protein by CDD hypothetical protein AND_11391 [*Anopheles darlingi*]	0.00120	E3X1K4
	gi|157019918|gb|EDO64520.1|	14-3-3 protein zeta AGAP007643-PA [*Anopheles gambiae* str. PEST]	0.00001	A0NBC2
	gi|157017258|gb|EDO64137.1|	conserved hypothetical protein AGAP004349-PA [*Anopheles gambiae* str. PEST]	0.07200	A7UT27
	gi|157019090|gb|EAA06067.5|	conserved hypothetical protein AGAP003775-PA [*Anopheles gambiae* str. PEST]	0.06400	Q7PS70
	gi|83016748|dbj|BAE53441.1|	DsRed [synthetic construct]	0.01400	Q2WG74
	gi|157014263|gb|EAA43629.4|	adenylyl cyclase-associated protein AGAP010175-PA [*Anopheles gambiae* str. PEST]	0.00320	Q7PJT7
	gi|157017970|gb|EAA07771.4|	multiple Ig-domain containing protein by CDD AGAP002737-PA [*Anopheles gambiae* str. PEST]	0.00330	Q7QCP0
	gi|157017285|gb|EAU76851.2|	nucleoplasmin superfamily AGAP004395-PA [*Anopheles gambiae* str. PEST]	0.03700	A0NDN1
**Under-represented proteins in myrAkt ** ***An. stephensi***				
Epithelial integrity	gi|347970325|gb| EAA44666.5	perlecan AGAP003656 [*Anopheles gambiae* str. PEST]	0.0052	F5HLD4
	gi|19848250|gb| AAL99382	collagen IV alpha I chain fragment [*Anopheles gambiae*]	0.0021	Q8T7S4
	gi|19848250|gb| EAA10481.4	laminin A AGAP004993 [*Anopheles gambiae* str. PEST]	0.011	Q7PPF9
Chromatin integrity	gi|55234461|gb| EAA00131.2	histone H2B AGAP012199 [*Anopheles gambiae* str. PEST]	0.00068	Q27442
	gi|312384904|gb| EFR29519.1	histone H3 AND_23756 [*Anopheles darlingi*]	0.0079	E3WLC5
	gi|158298153|gb| XP_318361.3	histone H4 AGAP003909 [*Anopheles gambiae* str. PEST]	0.00012	B1Q2A0
Metabolism	gi|157015094|gb| EAA12479.4	**dihydrolipoamide acetyltransferase AGAP007975 [** ***Anopheles gambiae*** ** str. PEST]**	**0.0000028**	**Q7Q3P5**
	gi|157014514|gb| EAA13250.3	4-hydroxyphenylpyruvate dioxygenase AGAP004802 [*Anopheles gambiae* str. PEST]	0.018	Q7Q2T3
	gi|333469601|gb| EAA13629.5	alpha glucosidase AGAP003993 [*Anopheles gambiae* str. PEST]	0.00029	Q7Q275
	gi|166215094|gb| Q7PPA5.5	calcium-transporting ATPase sarcoplasmic/endoplasmic reticulum type AGAP006186 [*Anopheles gambiae* str. PEST]	0.00037	Q7PPA5
	gi|157020374 |gb| EAA04524.4	nucleoside diphosphate kinase AGAP007120 [*Anopheles gambiae* str. PEST]	0.025	Q7QIX6
Salivary gland	gi|124244265 |gb| ABM92299.1	salivary defensin [*Anopheles stephensi*]	0.025	A2TJI3
	gi|29501530|gb| AAO74842.1	gSG6 salivary gland protein [*Anopheles stephensi*]	0.0067	Q86M93
Other	gi|333467703|gb| EAA07856.5	EGF-like domain containing protein by CDD AGAP003027-PA [*Anopheles gambiae* str. PEST]	0.0062	Q7PR44

1 As of 9 September 2012.

The overrepresentation of proteins from Krebs' cycle and the electron transport chain (ETC) was interpreted as increased mitochondrial biogenesis to enhance oxidative phosphorylation (OXPHOS) output and perhaps to replace oxidatively-modified mitochondrial proteins. An increase in OXPHOS output would be consistent with elevated levels of the alpha subunit of Na^+^/K^+^-ATPase (**Table 1**). This enzyme, known to be located in the basolateral plasma membrane close to mitochondria-enriched fractions [39], catalyzes the hydrolysis of ATP coupled with the exchange of Na^+^ and K^+^ across the plasma membrane creating an electrochemical gradient, which sustains the resting membrane potential as well as provides the energy for active transport of various nutrients [40], and is one of the major ATP-consuming units of the cell [30].

Among the 14 proteins that were underrepresented in myrAkt *An. stephensi*, 7 proteins (50%; **Table 1**) were associated with epithelial and chromatin integrity. In particular, perlecan, collagen, and laminin – key components of the extracellular matrix (ECM) – were underrepresented in HM and HT *An. stephensi*. ECM proteins can be degraded and fragmented by oxidative stress [41], [42], suggesting that ECM/epithelial integrity was compromised in myrAkt *An. stephensi*. Histones H2B, H3, and H4 were also underrepresented in myrAkt *An. stephensi* relative to NTG mosquitoes. Histones are essential for chromatin packaging and DNA damage has been identified as a major mediator of chromatin reorganization and histone loss [43], suggesting that oxidative damage to DNA in myrAkt *An. stephensi* at 3–5 d post-emergence was likely to be high.

Four cytoskeletal proteins were overrepresented in myrAkt *An. stephensi*, including orthologs of microtubule-associated protein 1A and myosin light chain, proteins that are critically associated with mitophagy and autophagy, respectively [44], [45]. A third cytoskeletal protein, troponin T, a member of the troponin complex, regulates the interaction of myosin light chain with actin via tropomyosin, and, therefore, is associated with actin-myosin contractility. Mutations in troponin T in mice have been associated with mitochondrial degradation and the formation of increased numbers of small, round mitochondria with loss of well-defined membranes and cristae [46], indicating the importance of this protein in mitophagy and mitochondrial structure and function. Spectrin was also overrepresented in HM and HT *An. stephensi* relative to NTG females. Changes in levels of spectrin, a protein that links the actin cytoskeleton to the plasma membrane, precede autophagic cell death in salivary glands of *D. melanogaster* [47].

**Figure 1:**
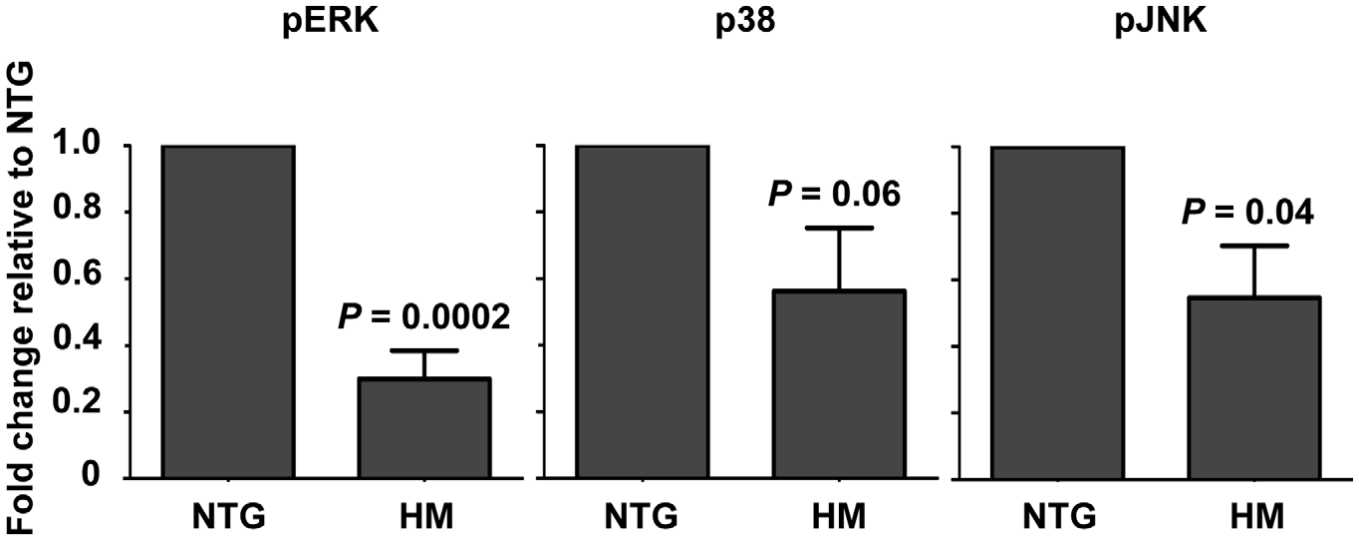
Over-expression of myrAkt was associated with reduced MAP kinase activation in the *An. stephensi* midgut. Midguts from 3–5 day old female HM myrAkt and NTG *An. stephensi* were dissected at 0.5 h post blood feeding and processed for western blot. Data are represented as the average fold change ± SEM of phospho-protein levels for pERK, p-p38, and p-JNK quantified by densitometry and normalized first to GAPDH to control for protein loading differences and then to phospho-protein levels in NTG controls. Data collected from 6–8 separate cohorts of *A. stephensi* were analyzed by Student's t-test (alpha = 0.05). *P* values are noted on the graph.

The upregulation of cytoskeletal proteins associated with the progression of autophagy and mitophagy suggested some involvement of these processes in the phenotype of myrAkt *An. stephensi*. To address this hypothesis, we examined activation levels (phosphorylation) of autophagy-promoting ERK, JNK, and p38 MAPK in the midguts of 3–5 d old, age-matched NTG and HM *An. stephensi*. Activation levels of all three MAPKs were reduced in the midgut of HM females relative to NTG females (by 40–60%; **Fig. 1**), which in the context of Akt overexpression, suggested a state of disruption of normal, autophagic repair processes in the midgut epithelium. Together with our LC-MS/MS data, these data suggested that proteins associated with mitochondria and maintenance of the structure of the midgut were altered by tissue-specific Akt overexpression in *An. stephensi*.

### myrAkt overexpression resulted in abnormal midgut and mitochondrial morphologies

To investigate the impact of myrAkt overexpression on general midgut morphology and on midgut mitochondrial size, number, and shape, we completed morphometric analyses on TEM micrographs from posterior midguts of age-matched, 3 d and 18 d NTG, HT, and HM female *An. stephensi*. While the majority of blood digestion occurs in the posterior part of the midgut [48], this analysis provided the additional benefit of exploring a midgut region that that is enriched for endocrine cell-associated intestinal stem cells that are required for epithelial repair and maintenance [49]–[51]. The analyzed micrographs from the posterior midgut covered an average area of 462.9 µm^2^ or the equivalent of 5–8 cells per posterior midgut region. Each image section included an average of 72.9 µm of the midgut epithelium brush border and the average depth of the midgut sample from the edge of the brush border to the edge of the image was 85.9 µm.

**Figure 2:**
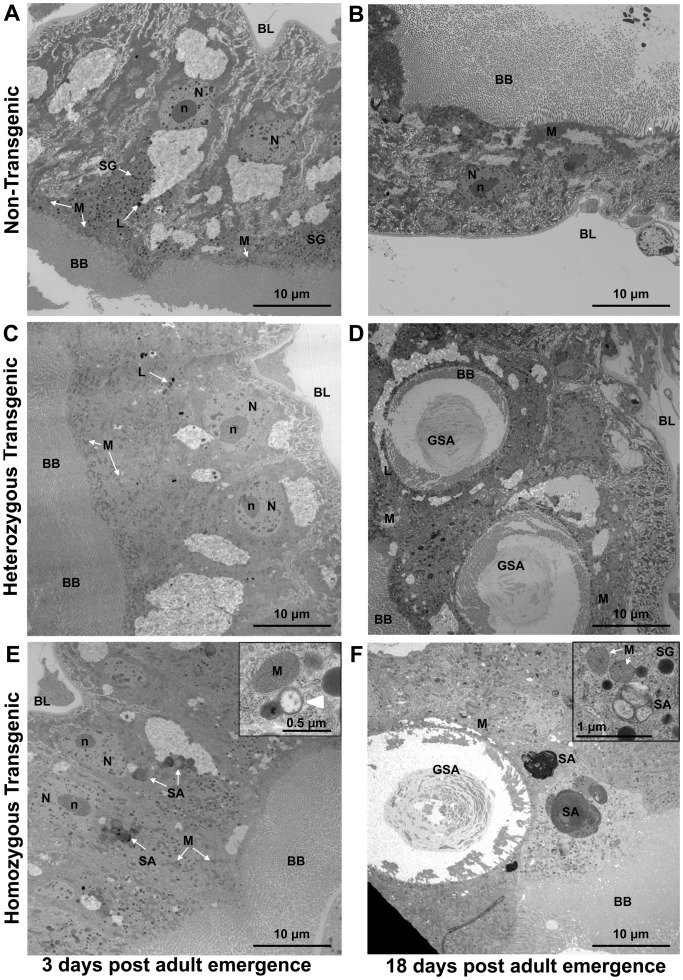
Over-expression of myrAKT led to morphological changes in the epithelium and mitochondria of the *An. stephensi* midgut. Posterior midgut morphology of NTG *An. stephensi* (3 d (A) and 18 d (B) after adult emergence), HT myrAkt *An. stephensi* (3 d (C) and 18 d (D) after adult emergence), and HM myrAkt *An. stephensi* (3 d (E) and 18 d (F) after adult emergence). Midguts from 3 d NTG, HT, and HM *An. stephensi* had an intact brush border (BB) and mitochondria (M) localized near the brush border (A, C, E). Stalled autophagosomes (SA) were observed in the midguts of 3 d HM (E) and 18 d HM and HT mosquitoes (D, F). Giant stalled autophagosomes (GSA) were found in the midguts of 18 d HM and HT mosquitoes (D, F). Many SAs contained electron dense material consistent with engulfed mitochondria (E, F). An example of a clear, double membrane, autophagic vacuole fusing with a small, apparently damaged, mitochondrion in the midgut of 3 d HM (white arrowhead, E inset) indicated mitophagy in process. In addition, the mitochondria in 18 d HM mosquitoes were not localized to the brush border, but distributed through the cytoplasm (F). An inset shows example of a stalled autophagosome containing three partially degraded mitochondria in an 18 d HT midgut (F inset). Representative TEM images taken at a magnification of 2,650× are shown. Inset in (E) is at magnification of 15,000×, and inset in (F) is at 8,800×. Midgut epithelium microvilli or brush border, BB; lysosomes, L; basal lamina mitochondria, M; cell nucleus, N; nucleolus, n; stalled autophagosomes, SA; giant autophagosomes with brush border inside, GSA; secretory granules, SG; white arrows point at representative structures.

The overexpression of myrAkt resulted in significant morphological changes to the *An. stephensi* posterior midgut (**Table 2**). Posterior midgut tissue of 3 d and 18 d NTG mosquitoes had a well-defined brush border, intact basal lamina, well-defined nuclei, normal lysosomes, and numerous mitochondria that tended to localize near the brush border (**Figs. 2A, B**) as observed in other insect species [52]–[54]. The midgut morphology of 3 d HT and HM females were similar to those of NTGs with most structures comparable to NTG mosquitoes. However, a significantly higher prevalence of midguts from 3 d transgenic mosquitoes had cytoplasmic inclusions (40% HT and 60% HM) that were less common or absent in midguts from NTG females (20%; **Table 2**). Based on morphology (outer double membrane around electron dense material, membrane-like structures, membrane-bound structures with cristae-like organization; [55], [56]), these inclusions were likely stalled autophagosomes of various sizes (**Figs. 2E**). Mitochondrial autophagy in particular was evident as either mitochondrial membranes engulfed by a developing phagolysosome or as autophagosome-associated mitochondrial degradation (**Fig. 2E, F** **insets**).

**Table 2:** Summary of morphological changes in the posterior midguts of non-transgenic (NTG), homozygous (HM), and heterozygous (HT) myrAkt *An. stephensi* females at 3 d and 18 d post emergence.

Genotype	Age	Brush Border (BB) damage	Mitochondria localized to the BB	Proportion of midguts with Stalled Autophagosomes (SA)*	Proportion of midguts with giant SAs with BB inside
NTG	3 d	No	Yes	1/5	0
HT	3 d	No	Yes	2/5	0
HM	3 d	No	Yes	3/5	0
NTG	18 d	No	Yes	1/4	0
HT	18 d	Yes	Yes	3/4	2/4
HM	18 d	Yes	No	3/4	4/4

* Under this category, we included vacuoles with electron dense content and small and large autophagosomes with membrane material (e.g., membrane, mitochondrial remnants). To count a midgut as containing autophagosomes, two 95% confidence intervals (CI) were constructed using NTG values at 3 and 18 d ([4.4, 17.9] and [7.7, 33.8]). The numerator indicates the number of midguts that contained a number of autophagosomes above the highest 95%CI limit. The denominator indicates the number of midguts evaluated for each condition. Using the Chi-square test, NTG versus HT, NTG versus HM, and HT versus HM were significantly different at *P*<0.002.

**Figure 3:**
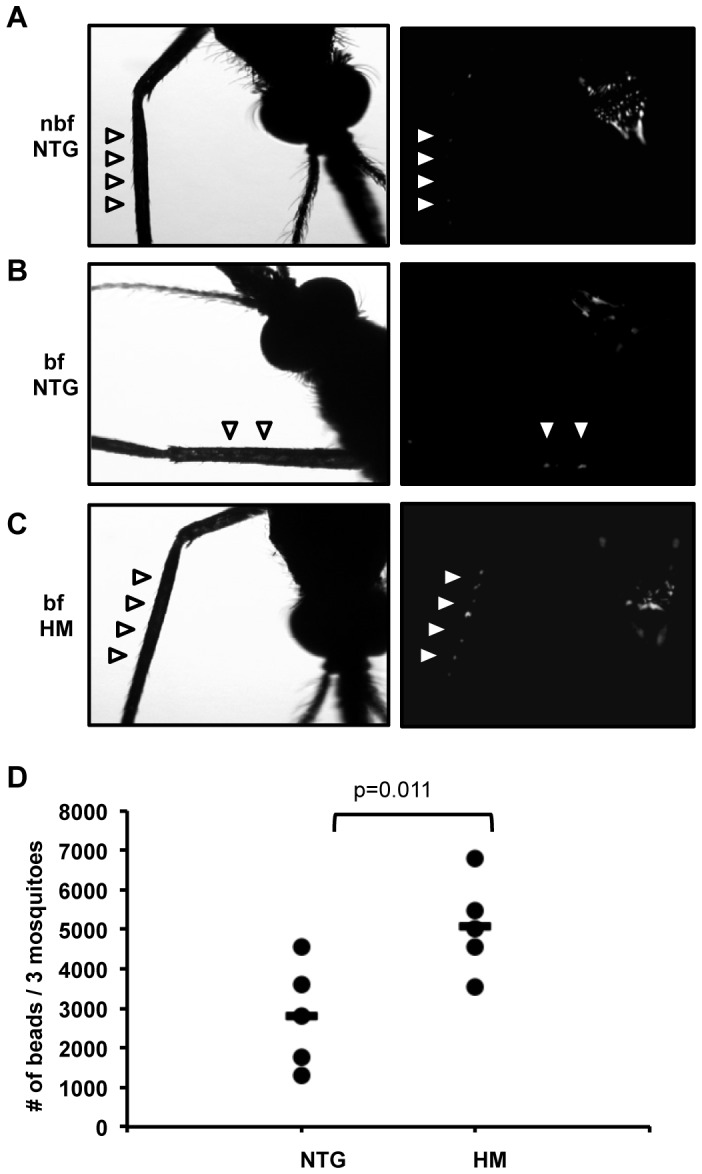
Evaluation of midgut permeability in HM myrAkt relative to NTG *An. stephensi*. (A) 3–5 d old, age-matched non-blood-fed NTG (nbf NTG), (B) blood-fed (bf) NTG and (C) HM female mosquitoes, were fed fluorescent beads (3–3.5 µM, Spherotech) in a reconstituted human blood meal through a Hemotek Insect Feeding System (Discovery Workshops). Prior to feeding (nbf NTG) and at 48 h post-blood feeding (bf NTG, bf HM), five whole mosquitoes from each group were cold-anesthetized and placed in 24-well plates. Mosquitoes were photographed under normal light (left panels) and also imaged on a Nikon TE 200 inverted fluorescent microscope at 4× with identical settings for all fluorescent images (right panels). Autofluorescence is visible on the mosquito thorax in all images. Arrowheads in nbf NTG mark location of mosquito leg and in bf NTG and bf HM images mark fluorescent beads in mosquito legs, an indication of bead passage through the midgut epithelium into the hemocoel. Note larger number of beads in bf HM compared to bf NTG *An. stephensi*. (D) Bead numbers per three whole mosquitoes minus bead numbers in three paired midguts from the same groups at 48 h post-feeding are represented as individual dots (means indicated as bars). Midgut beads averaged 236 for bf NTG and 225 for bf HM at 48 h post-feeding, so midgut beads accounted for less than 10% and less than 5%, respectively, of NTG and HM whole body bead counts. Data were analyzed by Student's t-test (alpha = 0.05).

Midgut epithelium is a highly aerobic tissue and deficits in energy from mitochondria, perhaps associated with altered mitophagy, would likely result in electrolyte imbalance, defective secondary active transport of cations and solutes, and increased permeability [57]. To determine whether histological changes observed in HM midguts were accompanied by altered epithelial integrity, 3–5 d old NTG and HM mosquitoes were fed with fluorescent beads in reconstituted human blood meals. After 48 h – to allow complete digestion of the blood – bead numbers were quantified by flow cytometry in washed, lysed midguts and in whole mosquitoes to estimate transport of beads across the epithelium. Body bead counts (minus midgut beads) from NTG females were 2,805±593 (mean ± SEM), whereas body bead counts from HM females were nearly 2-fold higher (5,073±534; *P* = 0.011; **Fig. 3**). Therefore, the permeability of the midgut epithelium was significantly increased in 3–5 d old HM females compared to age-matched NTG females, confirming that observed histological changes in the midgut epithelium of 3–5 d old HM myrAkt *An. stephensi* were functionally significant.

In 18 d NTG mosquitoes, midgut morphology was comparable to 3 d NTG mosquitoes although an increased number of lysosomes were present (**Fig. 2B**). However, profound changes were observed in midguts from 18 d HT and HM transgenic mosquitoes (**Figs. 2D, F**). In both HT and HM females we observed multiple giant stalled autophagosomes containing stacks of membrane-like material (**Figs. 2D, F** **; Fig. S1A**). Some giant autophagosomes contained brush border microvilli (**Figs. 2D, F** **; Fig. S1B**), which likely formed as a result of invagination of brush border membrane (**Fig. S1B**). These giant stalled autophagosomes with remnants of brush border were present only in TG mosquitoes at 18 d (50% HT and 100% HM versus 0% NTG; **Table 2**). In addition, in midguts of 18 d HM females mitochondria were no longer localized to the brush border, but were instead evenly distributed throughout the cell cytoplasm (**Fig. 2F** **; Fig. S1B**).

**Figure 4:**
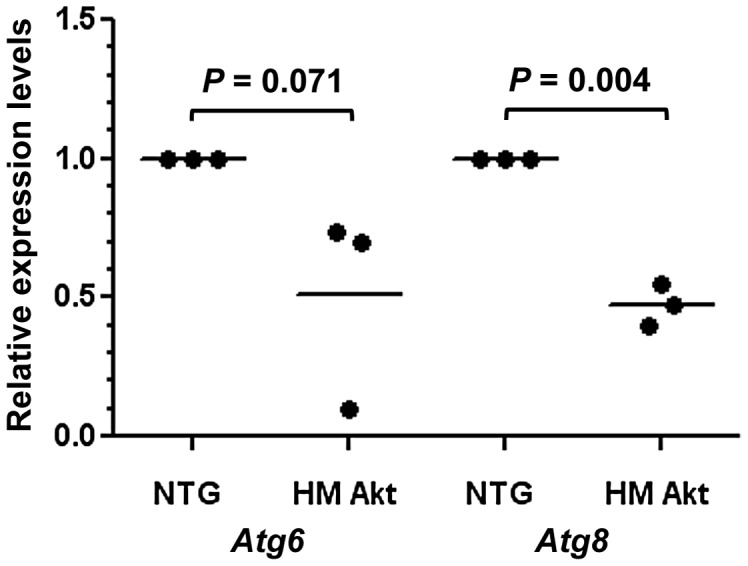
*Atg6* and *Atg8* expression levels were reduced in 18 d HM myrAkt relative to NTG *An. stephensi*. Midguts were dissected from 18 d HM myrAkt and NTG *An. stephensi* for RNA isolation and quantitative RT-PCR as described in the Methods. The analyses were performed on midgut RNAs from three independent cohorts of *An. stephensi*. Each data point represents *Atg6* or *Atg8* expression from one of three biological replicate samples; values were normalized to NTG levels (indicated as 1.0). Means are indicated as bars for each treatment. Data were analyzed by paired Student's t-test (alpha = 0.05) and *P* values are noted on the graph.

Although autophagy is required for normal mitochondrial turnover, the accumulation of inclusions suggested (i) stalled autophagy in HT and HM relative to NTG mosquitoes, (ii) normal autophagy overwhelmed by the high increase in damaged mitochondria, and (iii) over-reactive autophagy that could perhaps eliminate normal as well as dysfunctional mitochondria. In support of the first hypothesis, altered *Atg6* and *Atg8* mRNA expression levels were observed in the midgut of 18 d HM *An. stephensi* (**Fig. 4**). Atg6, also known as Beclin-1, is required for the generation of pre-autophagosome structures [58], while Atg8-phosphoethanolamine conjugates and the Atg5–Atg12 complex are essential components of the autophagosomal membrane [59]. In 18 d HM females, expression of *Atg8* was significantly reduced relative to NTG females (**Fig. 4**), while expression of *Atg6* showed a similar, non-significant trend, suggesting that autophagosome maturation is decreased in 18 d HM females.

**Figure 5:**
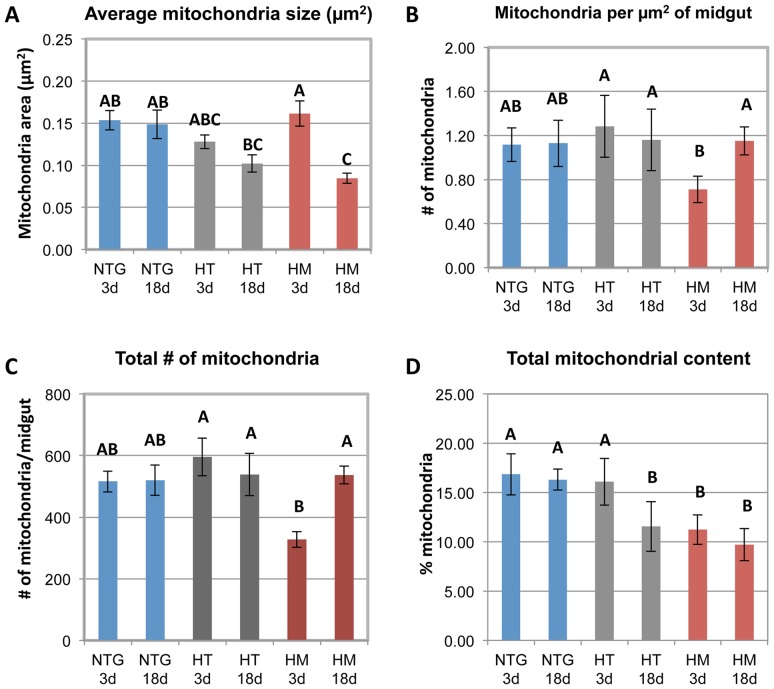
Over-expression of myrAKT was associated with changes in midgut mitochondria number and size. The number and size of mitochondria in the posterior midgut of myrAkt TG and NTG mosquitoes were determined for young (3 d) and old (18 d) mosquitoes within 85.90 µm of the brush border. (A) Average mitochondrial size was significantly decreased in midguts of 18 d old HM females compared to 18 d NTG and to 3 d HM. (B, C) The total number of mitochondria and mitochondrial density was significantly lower in 3 d HM mosquitoes relative to 3 d HT. In addition, the number and density of mitochondria significantly increased as the HM mosquitoes aged. (D) Total mitochondria content (percent area occupied by mitochondria) was significantly decreased in 18 d HT and HM mosquitoes and in 3 d HM mosquitoes relative to NTG controls and 3 d HT mosquitoes. Measurements from 5 midguts for each 3 d genotype and 4 midguts for each 18 d genotype were analyzed using a two-way ANOVA followed by Tukey-Kramer HSD test. Different letters indicate significant differences (alpha = 0.05).

Quantitative morphometric analysis of mitochondria in the posterior midgut revealed a significant decrease in the average size of mitochondria in midguts from 18 d HM females compared to similarly aged NTG females (by 47%, *P*<0.05; **Fig. 5A**). Midguts from 18 d HT also showed a decrease in average mitochondrial size (33% relative to NTGs) although this was not significantly different from 18 d NTG controls. In contrast to mitochondrial size, the number of mitochondria per µm^2^ of midgut and the total number of mitochondria per midgut decreased by 37% and 36% respectively in 3 d HM compared to NTG females; however, these decreases were not significant (**Figs. 5B, C**). The total of number of mitochondria per µm^2^ and the total number of mitochondria per midgut in 3 d HM were significantly lower than 3 d HT TG (45% decrease; *P*<0.05).

Total mitochondrial content – represented as a percentage of posterior midgut area occupied by mitochondria and based on mitochondrial number and size – was significantly lower in 3 d HM females compared to 3 d NTG females (by 33%, *P*<0.05; **Fig. 5D**), which was consistent with the reduction in mitochondria numbers in 3 d HM females (**Fig. 5C**). Interestingly, there was no corresponding decrease in the total mitochondrial content of 3 d HT females relative to 3 d NTG females. However, at 18 d after adult emergence, midguts from both HT and HM females had a significant reduction in total mitochondrial content relative to NTG females (by 29% in HT, *P*<0.05; by 40%, *P*<0.05 in HM; **Fig. 5D**). These differences were reflective of reductions in size of mitochondria in midguts of 18 d HT and 18 d HM females (**Fig. 5A**).

**Figure 6:**
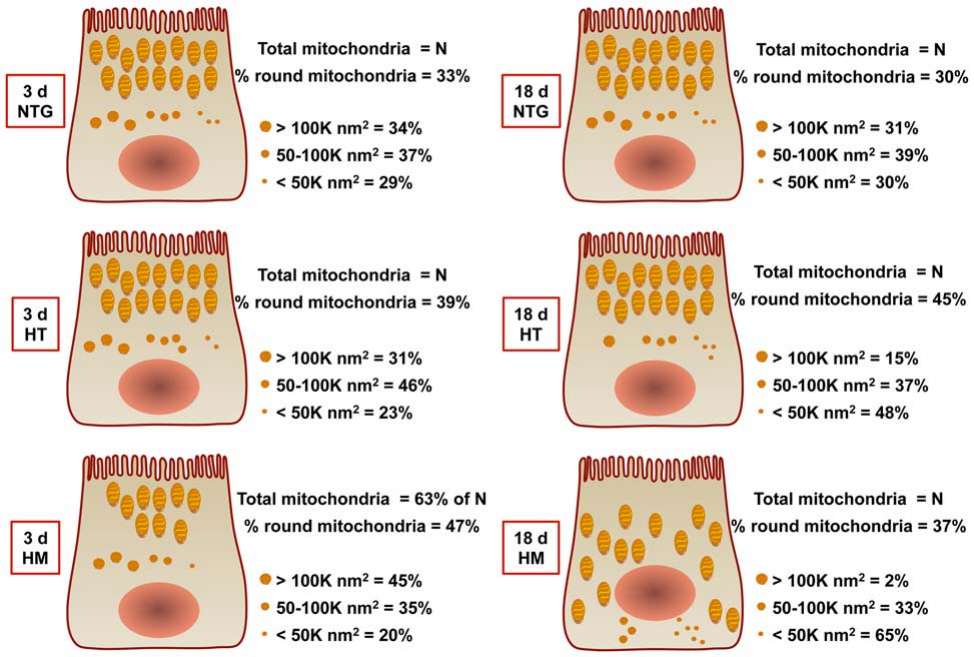
Over-expression of myrAKT was associated with changes suggestive of stalled mitochondrial fission. No differences were observed for percentages of round midgut mitochondria among NTG, HT, or HM myrAkt transgenic *An. stephensi* at 3 d and 18 d post-emergence (ANOVA following arcsin transformation; alpha = 0.05). In all groups except for 18 d HM, midgut mitochondria were associated with the brush border. In 18 d HM, midgut mitochondria were distributed throughout the cell. Further, size distributions of round mitochondria in NTGs were significantly different from those in HT and HM midguts at 3 d (NTG vs HT: χ^2^ = 20.5, df = 2, *P*<0.0001; NTG vs HM: χ^2^ = 19.6, df = 2, *P*<0.0001) and at 18 d (NTG vs HT: χ^2^ = 54.8, df = 2, *P*<0.0001; NTG vs HM: χ^2^ = 244, df = 2, *P*<0.0001). In addition, within each TG genotype (HT and HM), there were significant differences in size distributions of round mitochondria between samples analyzed from 3 d and 18 d *An. stephensi* (3 d HT vs 18 d HT: χ^2^ = 182, df = 2, *P*<0.0001; 3 d HM vs 18 d HM: χ^2^ = 457, df = 2, *P*<0.0001).

The overrepresentation of mitochondrial proteins (3–5 d; **Table 1**), the accumulation of autophagosomes (3 d HM and HT; **Table 2**), apparent altered autophagy (18 d HM; **Fig. 4**), and changes in size, number, and distribution of mitochondria in the midguts of 3 d HM (**Fig. 5D** **,** **Table 2**) and 18 d HT (**Fig. 5D**) *An. stephensi* were indicative of defective organelle maintenance in TG mosquitoes. Accordingly, we quantified the percentages of round versus non-round mitochondria to assess the balance of fission-fusion with the assumption that functional midgut mitochondria exhibit a tubular, elongated shape while round mitochondria form in cells undergoing a response to cellular oxidative damage. We examined the shape of 2524 mitochondria in 3 d NTG, 2979 in 3 d HT, 1638 in 3 d HM, 2080 in 18 d NTG, 2154 in 18 d HT, and 2147 mitochondria in 18 d HM *An. stephensi*. The number of midgut epithelial cells from which we analyzed mitochondrial shape could not be determined for 18 d HT and 18 d HM mosquitoes due to extensive tissue damage induced by transgene expression in older mosquitoes. Thus, mitochondria were counted over an identical midgut area for all treatments. As expected, a high percentage of elongated, tubular mitochondria were observed in NTG midguts (**Fig. 6**). Contrary to our expectations, however, no significant differences in the percentages of round mitochondria among midguts of NTG, HM, and HT females at 3 d or 18 d or between matched genotypes at 3 d or 18 d were observed (*P* = 0.088). However, analysis of the distributions of small (<50,000 nm^2^), medium (50,000–100,000 nm^2^), and large (>100,000 nm^2^) round mitochondria showed significant differences between and among NTG, HT, and HM females. The distributions of small, medium, and large round mitochondria were comparable in midguts from 3 d and 18 d NTGs (**Fig. 6**). However, the distributions of round mitochondria in NTGs were significantly different from those in HT and HM midguts at both 3 d and 18 d (*P*<0.0001). In addition, within each transgenic genotype (HT or HM), there were significant differences in mitochondrial size distributions between samples analyzed from 3 d and 18 d *An. stephensi* (*P*<0.0001). At 18 d, the occurrence of small, round mitochondria showed a gene-dose dependence from NTG to HT to HM females (30% to 48% to 65%; **Fig. 6**). The increased percentages of small mitochondria were accompanied by losses in both medium and large sized mitochondria, with percentages of both sizes trending downward from NTG to HT to HM females at 18 d. These changes in morphology appeared to be consistent with persistence of oxidative stress-induced fission, resulting in the formation of small, round mitochondria that can persist when fusion is inhibited [60]–[63].

### myrAkt overexpression was associated with reduced midgut mitochondrial function, enhanced nitrative damage

Despite obvious abnormal mitochondrial morphology, our LC-MS/MS data indicated that mitochondrial proteins were overrepresented in midguts of myrAkt transgenic *An. stephensi*. Successful mitochondrial biogenesis requires a correct sequence of events consistent with increased and coordinated synthesis of mitochondrial precursors from the nuclear and mitochondrial genomes, followed by import and assembly of nuclear-encoded subunits. Importantly, if any processes downstream from overexpression of mitochondrial proteins (including post-translational processes such as increased RNOS-mediated stress) are altered, then mitochondrial OXPHOS would be compromised despite an available excess of individual subunits. To evaluate this possibility, individual Complex activities were evaluated in whole midguts from 3 d and 18 d NTG and HM *An. stephensi* females along with citrate synthase, a marker for mitochondrial mass [8] (**Table 3**). Citrate synthase activity in midguts from HM females was approximately 10% higher than NTG females at 3 d. This value contrasted with the 33% reduction in posterior midgut mitochondrial content of 3 d HM females by morphometric analyses (**Fig. 5D**). By 18 d, citrate synthase was decreased by 40% in the whole midgut, consistent with the 40% reduction in total mitochondrial content revealed by morphometric analyses of posterior midgut (**Fig. 5D**). The discrepancy at 3 d could be due to spatial changes in mitochondrial mass over time in the midgut. In particular, mitochondrial loss may be evident at 3 d only in the posterior region of the HM midgut, which was examined morphometrically, but not detectable biochemically at the level of the whole midgut at this time. By 18 d, changes in total mitochondrial content/mass may have spread from the posterior region throughout the midgut, as revealed by concordance between poster midgut morphometrics and whole midgut biochemical analyses.

**Table 3:** Mitochondrial outcomes in midguts of non-transgenic (NTG) and myrAkt homozygous (HM) *An. stephensi* females at 3 d and 18 d post emergence.

	3 d				18 d			
Outcome	NTG	HM	*P* value	fold	NTG	HM	*P* value	fold
**Midgut/mg protein**	70.0±0.7	54.53±0.02^a^	0.002	0.8	69.3±0.8	67.3±3^a^	0.57	-
**Activities nmol (min×midgut)^−1^**								
**CS**	3.70±0.09^b^	4.16±0.05^c^	0.004	1.1	2.50±0.02^b^	1.40±0.07^c^	3×10^−6^	0.6
**Complex I (NQR)**	0.81±0.01^d^	0.77±0.05^e^	0.770	-	0.44±0.06^d^	0.09±0.02^e^	0.04	0.2
**Complex I (NFR)**	3.7±0.3	1.9±0.2	0.013	0.5	3.2±0.9	2.5±0.5	0.049	0.8
**Complex II–III**	0.81±0.05	0.27±0.04	0.002	0.3	0.44±0.08	0.18±0.06	0.045	0.4
**Complex V**	10.1±0.3	6.9±0.3^f^	2×10^−4^	0.7	9.7±0.8	4.6±0.2^f^	7×10^−4^	0.5
**Activities normalized to CS**								
**Complex I (NQR)**	0.220±0.003	0.19±0.01^g^	0.121	-	0.18±0.02	0.07±0.01^g^	0.050	0.4
**Complex I (NFR)**	1.00±0.09	0.47±0.04^h^	0.008	0.5	1.6±0.2	1.6±0.4^h^	0.992	-
**Complex II–III**	0.19±0.03	0.08±0.02	0.027	0.4	0.21±0.01	0.09±0.01	0.005	0.4
**Complex V**	2.74±0.08^i^	1.66±0.07^j^	5×10^−5^	0.6	3.9±0.3^i^	3.4±0.1^j^	0.17	-
**Non-mitochondrial ATPases nmol×(min×midgut)^−1^**	9.3±0.4^k^	12.8±0.5^l^	0.001	1.4	4.6±0.04^k^	3.8±0.05^l^	2×10^−5^	0.8
**NOS activity/midgut** *	1.0	1.75±0.05	0.031	1.8	nd	nd		
**NitroTyr (OD/midgut)**	0.4±0.1^n^	3.1±0.2^o^	0.002	8	3.8±0.3^n^	6.6±0.6^o^	0.009	1.7
**NitroTyr/beta subunit**	0.43±0.02^p^	1.27±0.10^q^	0.001	3	0.08±0.01^p^	0.14±0.02	0.049	1.8
**ECP (midguts)**	0.80±0.02	0.75±0.09	0.05	0.94	0.80±0.01	0.71±0.05	0.05	0.89

Identical superscripts indicate values significant differences with the following *P* values: (a) 0.001, (b) 1×10^−5^, (c) 5×10^−8^, (d) 2×10^−3^, (e) 1×10^−4^, (f) 8×10^−4^, (g) 5×10^−4^, (h) 0.03, (i) 0.01, (j) 4×10^−5^, (k) 4×10^−5^, (l) 2×10^−6^, (m) 8×10^−5^, (n) 1×10^−4^, (o) 0.043, (p) 2×10^−5^, (q) 5×10^−4^; CS = citrate synthase; nd = not determined;

* defined as EPR area of spin adduct MGD-NO obtained under optimal conditions for NOS activity (meaning maximum NO produced by NOS, but not the NO produced by each type of mosquito after 3 d).

**Figure 7:**
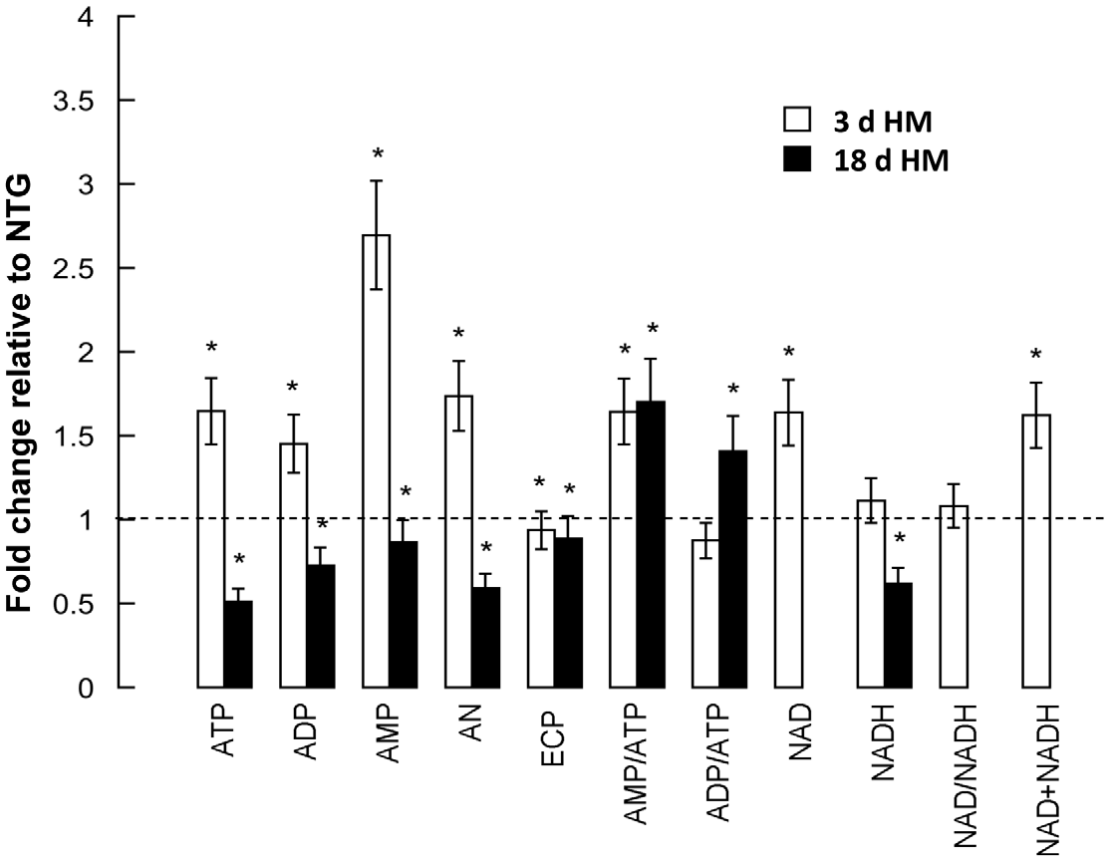
Midgut metabolite profiles in HM myrAkt *An. stephensi* suggested marked energy deficiencies relative to NTG mosquitoes at 3 d and 18 d post-emergence. All metabolites were evaluated by HPLC as described under Methods. ATP, ADP, AMP, AN (ATP+ADP+AMP), NAD, NADH and NAD+NADH were calculated as nmol/midgut and data are represented as fold of NTG values. The NTG values were the average of 3 d and 18 d given that no statistical differences were observed between these two days. Data were analyzed relative to NTG values with Student's t-test (alpha = 0.05).*P<0.05 relative to NTG values.

Examination of OXPHOS capacity revealed that in HM mosquitoes, activities of Complex I, Complex II–III and Complex V were 75% (for Complex I average of both NQR and NFR activities), 30% and 70% of NTG controls at 3 d, respectively, whereas at 18 d, these values were 50%, 40% and 50% of NTG controls, respectively (**Table 3**). These activities were still lower than controls when normalized to citrate synthase, making them independent of the number of mitochondria present at any given time point in whole midguts (**Table 3**). Relatively lower ETC activities can result in lower OXPHOS and energy deficits. To test this hypothesis, the energy charge potential (ECP) – defined as ([ATP]+0.5*[ADP])/([ATP]+[ADP]+[AMP]; [64]) – was evaluated in NTG and HM female midguts at 3 d and 18 d post-emergence and in whole bodies of 3 d NTG and HM females. In midguts from HM females at 3 d, the ECP showed some decline (94% of controls), which was clearly lower at 18 d (89% of controls; **Table 3** **,** **Fig. 7**). In addition to local tissue effects, overexpression of myrAkt in the *An. stephensi* midgut was associated with significantly reduced whole body total adenosine metabolites. In particular, [ADP] (in percentage of total nucleotides) at 3 d was 2-fold higher, [AMP] was 7.7-fold higher, and ECP was significantly reduced relative to age-matched NTG controls (**Table 4**) despite the fact that Akt overexpression was targeted to the midgut of *An. stephensi*. These results (decreased ECP, higher [ADP]/[ATP] and [AMP]/[ATP] ratios) indicated clear energy deficiencies locally in the midgut and systemically in the body of HM mosquitoes, suggesting that (i) mitochondrial biogenesis could not be correctly completed (defects at import/assembly) or (ii) oxidative/nitrative stress-mediated damage overpowered this putative compensatory response.

**Table 4:** Nucleotide contents in whole bodies of non-transgenic (NTG) and myrAkt homozygous (HM) female *An. stephensi* at 3 d post-emergence.

Metabolite	Concentration (nmol/mosquito)	
	NTG	HM
ATP	11.5±0.8	2.9±0.4*
ADP	1.59±0.09	0.85±0.05*
AMP	0.022±0.002	0.044±0.003*
Total nucleotides	13.1±0.4	3.4±0.4*
ECP	0.94±0.02	0.88±0.09*
ATP/ADP	7.2±0.6	3.4±0.8*
AMP/ATP	0.002±0.000	0.015±0.002*

* Different from controls with *P*≤0.05. ECP = energy charge potential.

**Figure 8:**
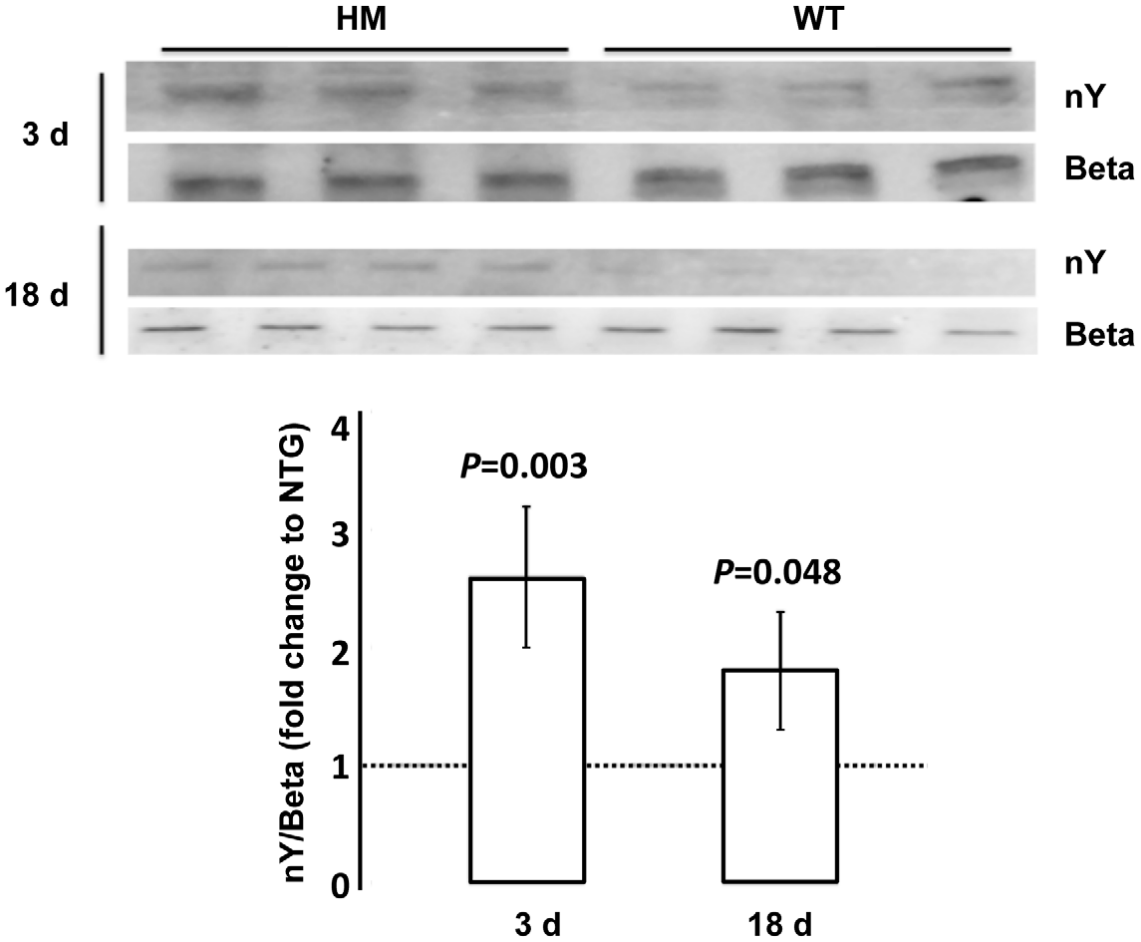
Tyrosine nitration of midgut ATPase beta subunit was increased in HM myrAkt An. stephensi relative to NTG females at 3 d and 18 d post-emergence. Top panel, representative western blots probed for nitrotyrosine (nY) and total ATPase beta subunit (Beta) of midgut proteins from HM and NTG *An. stephensi* at 3 d and 18 d post-adult emergence. Bottom panel, quantified ECL signals for nY were normalized to total ATPase beta subunit and represented as fold change relative to NTG *An. stephensi* (indicated as dotted line at 1.0). Data were analyzed using Student's t-test (alpha = 0.05); calculated *P* values indicate significant differences in levels of nitration of midgut ATPase beta subunit between age-matched midguts of HM and NTG *An. stephensi*.

**Figure 9:**
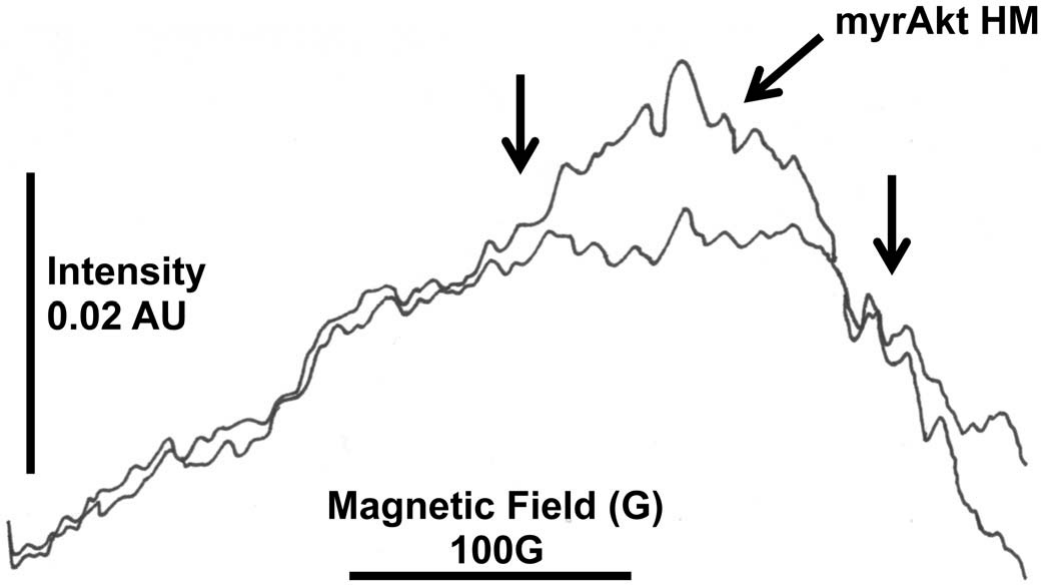
EPR detection of NO indicated marked NOS catalytic activity in midguts from 3 d HM myrAkt *An. stephensi* relative to 3 d NTG mosquitoes. Whole midguts from 150 NTG and 150 HM *An. stephensi* at 3-d post-adult emergence were each hand-homogenized in 300 µl of 20 mM HEPES, pH 7.4 with protease inhibitors and phosphatase inhibitors), then incubated for 3 h at 20–22°C following addition of 100 µl reaction buffer (3 mM sodium *N*-methyl-D-glucamine dithiocarbamate [MGD] complexed with ferrous sulfate prepared fresh), 0.1 mM NADPH, 1 mM calcium chloride and 1 mM L-arginine in degassed 20 mM HEPES, pH 7.4; [104]. After the incubation, 50 to 100 µl of sample was loaded into an EPR tube and measured using a Bruker EPR and XEpr software. Instrument conditions were indicated under Methods. X-band EPR analysis at 190°K of midgut homogenates produced a strong, broad EPR signal with resonance positions (g tensor factors) at g = 2.04 (left arrow) with an associated triplet signal at g = 2.014 with hyperfine splitting of 17.5 Gauss (right arrow). The broad paramagnetic signal at g = 2.04 has been attributed to the trapping of nitric oxide by NMGD-Fe resulting in the formation of NMGD-Fe-NO adduct. The area and/or amplitude of the adduct signal (indicative of concentration of free radicals, in this case NO) was markedly increased in TG midguts.

To test these possibilities, oxidative/nitrative damage was assessed by evaluating Tyr nitration of the beta subunit of ATPase (ATPB; **Fig. S2**), a sensitive marker for mitochondrial protein nitration [30], [31], given that Complex V activity was significantly lower than controls (50 to 70% of NTG). NOS activity was evaluated by detecting NO using electron paramagnetic resonance in conjunction with spin trapping technique. Nitration of midgut proteins in HM females was significantly increased at 3 d (8-fold of controls; *P* = 0.02) and at 18 d (2-fold of controls; *P* = 0.05) while nitration of mitochondrial ATPB was 3-fold (*P* = 0.003) and 2-fold (*P* = 0.048) of controls at each time point (**Table 3** **,** **Fig. 8**). The production of NO was 2-fold of controls at 3 d (**Table 3** **,** **Fig. 9**). Thus, nitrative/oxidative stress was enhanced in midguts from HM *An. stephensi* – even at 3 d post-emergence – consistent with the increased ATPB nitration and activity loss [65]. Further, NO is an inhibitor of Complex IV [66], [67] through competitive and noncompetitive pathways [66], suggesting increased NO production could block electron transport at the terminal oxidase, even when no changes in activity are detected, enhancing the RNOS-mediated damage of individual Complexes and/or mitochondrial targets and negating compensatory biogenesis.

While these data provided clear indications of mitochondrial dysfunction in myrAkt *An. stephensi* even in the absence of infection, they did not provide a mechanistic explanation for inhibition of *P. falciparum* development in myrAkt *An. stephensi* observed previously [5]. Collectively, our data suggested that inhibition of parasite development could result from (i) direct, toxic effects of RNOS on developing *P. falciparum* [68]–[70], (ii) indirect effects of RNOS-mediated mitochondrial dysfunction in the host (e.g., reduction in host energy required for parasite development, damage to midgut epithelial receptors/proteins required for parasite development), or (iii) some combination of these direct and indirect effects of Akt overexpression on developing parasites.

### Resistance to *P. falciparum* in myrAkt HM *An. stephensi* was reversed by NOS inhibition

**Figure 10:**
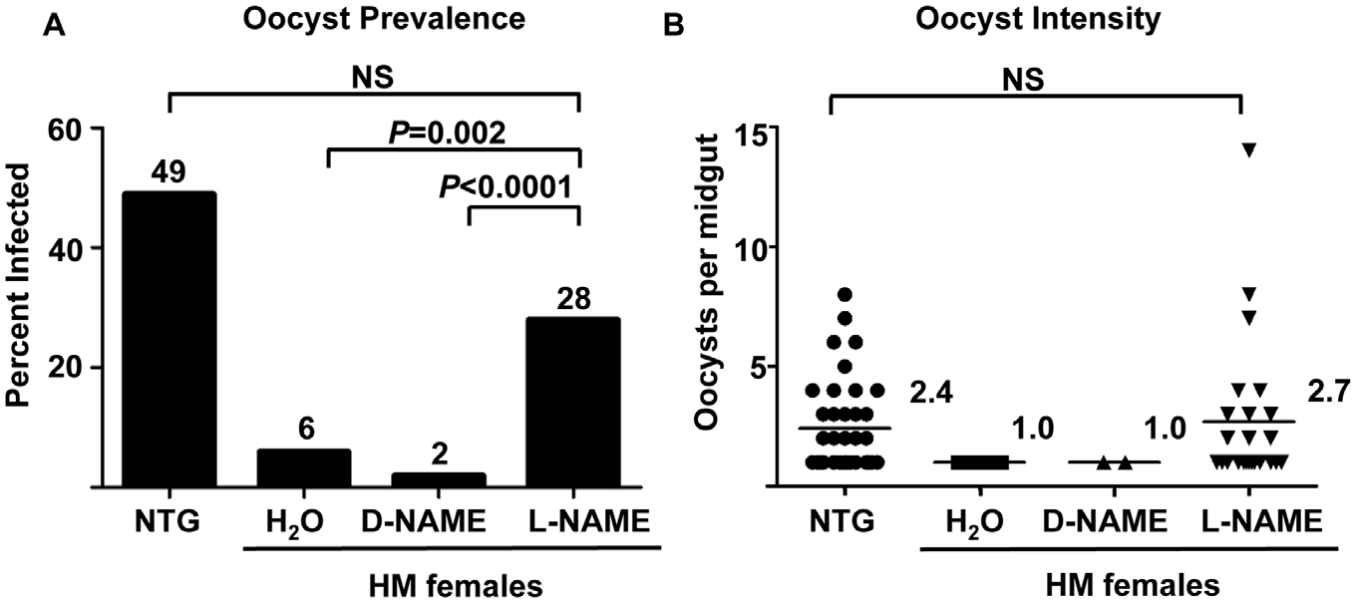
Resistance to *P. falciparum* in myrAkt HM *An. stephensi* was reversed by NOS inhibition. Preparation and treatments for mosquitoes is described in the Methods. (A) Prevalence of infection (percentage of mosquitoes dissected with at least one *P. falciparum* oocyst) of mosquitoes dissected in (B). Fisher's exact test was used to compare treatments against *L*-NAME; NS = not significant at alpha = 0.05. Numbers above bars reflect mean prevalences for control and treatment groups. (B) Water, *D*-NAME, *L*-NAME treatments with myrAkt HM *An. stephensi*. Age- and cohort-matched NTG control mosquitoes were infected side-by-side with the same parasite culture used for HM myrAkt *An. stephensi*. Numbers within the figure reflect mean oocysts per midgut for control and treatment groups. This experiment was repeated four times with four separate cohorts of mosquitoes. Data shown are from infected midguts (no zeros). H_2_O and *D*-NAME were outside of the 95% confidence intervals for NTG (1.79,3.05) and *L*-NAME (1.41,4.01); the latter groups were not different by Mann-Whitney U-test (alpha = 0.05).

To first assess whether overproduction of NO was responsible for resistance to *P. falciparum* in myrAkt *An. stephensi*, four separate cohorts of HM *An. stephensi* females were provided with water, *N* ^ω^-Nitro-*L*-arginine methyl ester (3.7 mM, *L*-NAME; [69], [71]) or the biologically inactive isomer *D*-NAME from 72 h before blood feeding though *P. falciparum* infection and thereafter until dissection. Age- and cohort-matched NTG females were infected side-by-side as controls. After 10 days, females were dissected to visualize and count *P. falciparum* oocysts. Among those NTG *An. stephensi* that were fully gravid (an indicator of complete engorgement), 49% had at least one midgut oocyst (**Fig. 10A**). Infected NTG mosquitoes averaged 2.4 *P. falciparum* oocysts per midgut (**Fig. 10B**). As expected, HM females provided only with water or with water with *D*-NAME were resistant to infection (**Fig. 10B**). However, provision of *L*-NAME to HM *An. stephensi* reversed the phenotype of resistance to infection, resulting in a prevalence and intensity of infection that were not significantly different from control, NTG females fed on the same *P. falciparum*-infected blood (**Figs. 10A, B**). Neither *L*-NAME nor *D*-NAME had significant effects on *P. falciparum* growth in the absence of the mosquito (**Fig. S3**). Although this growth assay cannot be performed efficiently on mosquito-stage parasites, we assert that our results suggest that observed infection patterns were due to *L*-NAME effects on the mosquito host.

**Figure 11:**
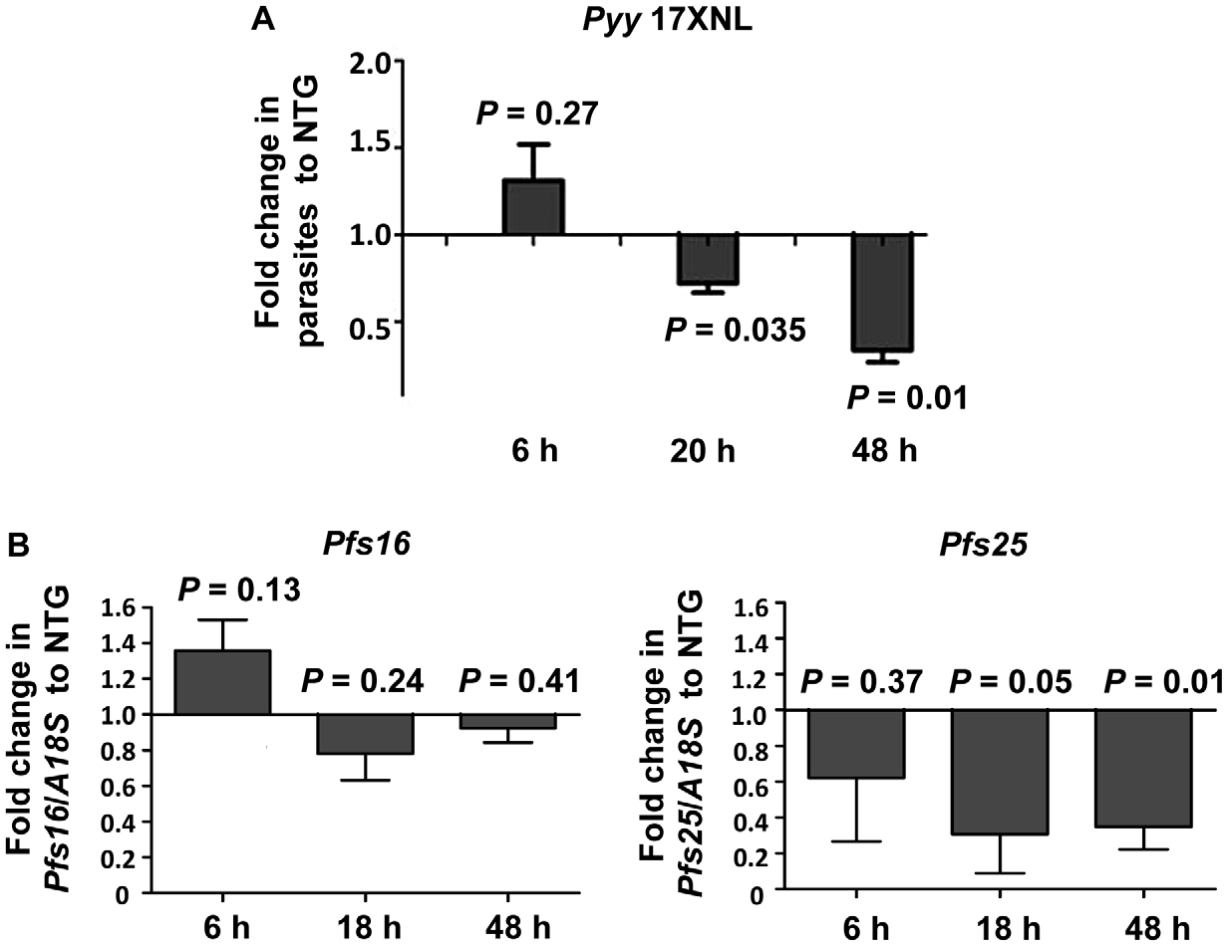
HM myrAkt *An. stephensi* exhibited enhanced killing of early stage GFP-*Plasmodium yoelii yoelii* 17XNL (Pyy 17XNL) and *P. falciparum* relative to NTG females. (A) Pyy 17XNL parasites were quantified in dissected midguts at 6, 20 and 48 h post-infection by fluorescence detection (485 nm excitation/535 nm emission wavelengths). Significant killing was noted by 20 h post infection, when Pyy 17XNL are present as fully matured ookinetes in the midgut lumen and in transit across the midgut epithelium [76]. A secondary drop in infection levels was evident at 48 h, which coincides with early oocyst development for Pyy 17XNL [76]. (B) *Plasmodium falciparum* parasites were quantified using real-time reverse-transcriptase PCR of *A18S* rRNA (for total parasites) as well as *Pfs16* and *Pfs25*, markers for sexual stage development [73], [74]. Changes in *Pfs16* expression were not significant but trended downward relative to expression in NTG *An. stephensi* at 18 h and 48 h post-infection. A reduction in *Pfs25* expression at 6 h suggested that early ookinetes were reduced in HM *An. stephensi*, with significant killing at 18 h post-infection, a time at which all ookinetes are still present in the midgut lumen [77]. A secondary significant drop was evident at 48 h, at which time ookinetes have traversed the midgut epithelium and are starting to transition to oocysts [77]. The data are represented as the average fold change ± SEM in the number of parasites for HM *An. stephensi* (black bars) compared to NTG *An. stephensi* at the same timepoints (indicated as the transition at 1.0). Data from independent experiments with three (A) or four (B) separate cohorts of *An. stephensi* females were analyzed by Student's t-test (alpha = 0.05) and *P* values are noted on the graph.

To determine whether the effects of NO were attributable specifically to direct, toxic effects on developing parasites, we examined more closely the timing of parasite death in HM females relative to parasite development in NTG *An. stephensi*. For these studies, we used a mouse malaria parasite infection model (GFP-expressing *Plasmodium yoelii yoelii* 17XNL; kindly provided by A. Rodriguez [72]) in addition to mosquito infection with *P. falciparum*. This design allowed us to examine mosquito infection using independent, quantitative measures and to determine whether NO-dependent parasite killing in myrAkt *An. stephensi* was unique to *P. falciparum* or more broadly effective against unrelated parasite species. Infection with *P. y. yoelii* was monitored using fluorescence quantitation, while *P. falciparum* infection levels were assessed with quantitative, reverse-transcriptase PCR for two markers of sexual stage parasite development, *Pfs16* and *Pfs25* [73]–[75]. In infections with both species of *Plasmodium*, significant parasite death was first observed by 18–20 h after infection (**Figs. 11A, B**). At 20 h post-infection, *P. y. yoelii* 17XNL parasites are present as mature ookinetes, with some in the midgut lumen and a large percentage in transit across the midgut epithelium [76]. At 18 h post-infection, all *P. falciparum* parasites are present only as ookinetes in the midgut lumen of *An. stephensi* [77]. A secondary significant drop in infection levels of HM *An. stephensi* relative to NTG females was observed by 48 h after infection for both parasite species – a time that coincides with ookinete to oocyst transition on the outside of the midgut epithelium for *P. falciparum* [77] and early oocyst development for *P. y. yoelii* [76] – suggesting that RNOS-mediated anti-parasite killing occurs over a broad period of parasite development. These data confirmed that anti-*P. falciparum* resistance in myrAkt *An. stephensi* is initiated as direct, early toxic effects of mosquito NO/RNOS on parasites prior to invasion of the midgut epithelium.

## Discussion

**Figure 12:**
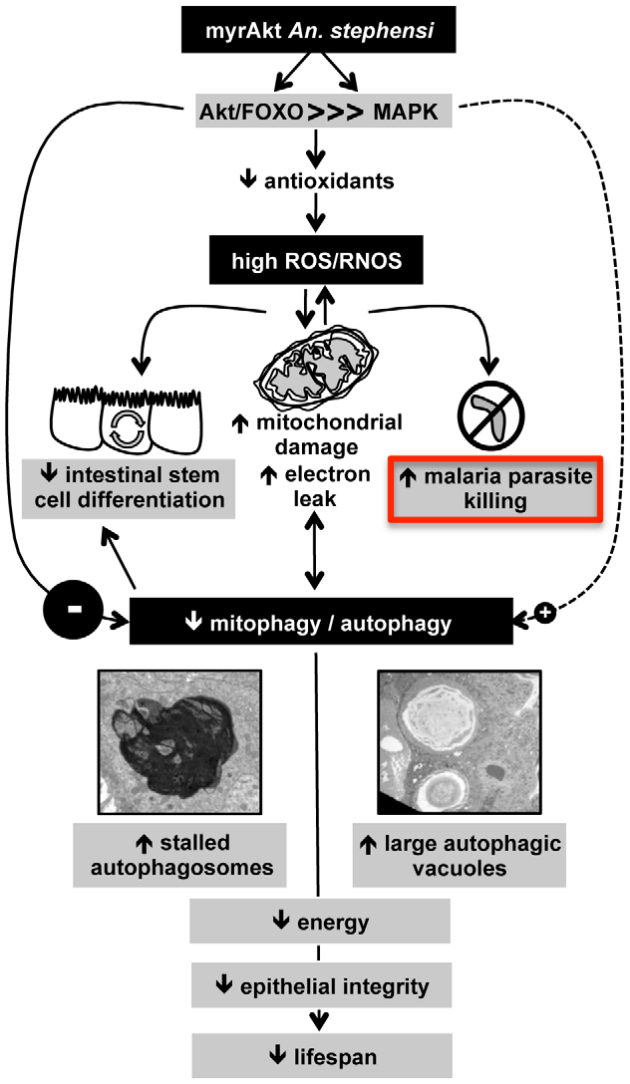
Over-expression of myrAkt in *An. stephensi* disrupts midgut mitochondrial dynamics, which impacts local and systemic physiology to drive anti-parasite resistance and reduced lifespan. The overexpression of myrAkt in the *An. stephensi* midgut induces FOXO phosphorylation [5], which results in a reduction in both mitochondrial and cytoplasmic antioxidants. This reduction leads to increased levels of ROS and RNOS, which lead to mitochondrial damage and a feedback cycle that enhances RNOS to levels that are necessary and sufficient for malaria parasite killing. Although oxidative damage is a strong activating signal for mitophagy, strong repressive signals from myrAkt overexpression and MAPK down-regulation prevent successful resolution of mitophagy, as evidenced by the accumulation of stalled autophagosomes. The incomplete resolution of mitophagy sustains oxidative stress, which results in local and systemic energy deficits, incomplete mitochondrial biogenesis, and epithelial damage that is unmitigated by autophagy-dependent stem cell differentiation. The balance between positive and negative signals for autophagy underlies autophagic regulation in and coordination of cellular homeostasis, epithelial barrier integrity, lifespan, and immunity [17]–[21]. As in other organisms, mitochondrial dysfunction, via Akt-dependent RNOS-mediated stress and dysregulated autophagy, perturbs midgut homeostasis or “midgut health” to enhance anti-parasite resistance and reduce the duration of mosquito infectivity.

Overexpression of a constitutively active Akt targeted to the midgut of *An. stephensi* inhibited *P. falciparum* infection and reduced the duration of mosquito infectivity [5]. Here, we have elucidated the mechanism for this process, demonstrating that Akt-dependent anti-parasite resistance is due to early, toxic effects of NO/RNOS followed by sustained mitochondrial dysfunction that cannot be mitigated via biogenesis and/or mitophagy/autophagy. Collectively, these phenomena lead to midgut epithelial damage (**Figs. 2** **, S1;** **Table 1**) and systemic energy deficiencies (as judged by ECP) that would be consistent with a reduction in lifespan and, as a consequence, a reduced infective lifespan. Activation of nuclear factor (NF)-kB-dependent immunity does not contribute to parasite resistance in myrAkt *An. stephensi*: Pakpour et al. [78] showed that activation of PI-3K-dependent signaling represses NF-KB activation in response to immune signals in *An. stephensi* cells *in vitro* and *in vivo*. Rather, overwhelming RNOS production with overexpression of myrAkt not only confers resistance to parasite infection, but also adversely impacts host infective lifespan. We would assert that balance and successful resolution of oxidative stress-induced mitophagy and mitochondrial biogenesis are the driving forces behind these phenotypes (**Fig. 12**) and that genetic manipulation of mitochondrial processes can provide a basis to alter multiple mosquito phenotypes to inhibit malaria parasite transmission.

In this study, we identified the molecular processes downstream of Akt overexpression in myrAkt *An. stephensi* that are involved in parasite infection resistance and reduction in lifespan. First, myrAkt protein levels in the midgut of transgenic *An. stephensi* increase significantly by 2 h after bloodfeeding and remain elevated above control levels through 12 h; these levels decline for the latter half of blood digestion and the reproductive cycle (24–48 h post-feeding; [5]). Hence, inducible overexpression of active Akt soon after blood ingestion would be available to repress FOXO-dependent antioxidants, including mitochondrial MnSOD, allowing a rise in damaging mitochondrial RNOS [27], [28]. We have previously demonstrated that toxic RNOS, which likely include peroxynitrite, can rise to high levels in the parasite-infected mosquito midgut after infection and that NOS-dependent killing is central to anti-parasite resistance in wild type mosquitoes [79]. However, the critical differences in the current studies are that Akt overexpression (i) leads to the overproduction of NO even in the absence of infection and (ii) represses cellular mitophagy, which would normally occur in response to infection-associated oxidative/nitrative stress, resulting in much more efficient parasite killing at a cost of both local and systemic energy synthesis and renewal/repair of the midgut epithelium that are essential for host survival following infection.

In addition to Akt-dependent signaling inhibition of mitophagy/autophagy, activated Akt controls mitochondrial biogenesis through phosphorylation of FOXO and exclusion of this transcriptional activator from the nucleus. In the absence of FOXO, there is no induction of PPAR-gamma coactivator-1 alpha (PGC-1a), a key mediator of mitochondrial biogenesis in mammalian cells [80]. Rera et al. [81] demonstrated that overexpression of FOXO-dependent *Drosophila* ortholog of PGC-1a led to an increase in abundance of respiratory complexes I, III, IV, and V and an increase in respiratory chain activity, indicating that control of biogenesis is conserved. Hence, in transgenic *An. stephensi*, activated Akt likely represses mitochondrial biogenesis through FOXO phosphorylation, which results in lower total mitochondrial content at 3 d and 18 d by morphometric analyses of the posterior midgut (**Fig. 5C**) and at 18 d as indicated by citrate synthase activity in the whole midgut (**Table 3**).

Despite the decrease in mitochondrial mass, we observed an upregulation of mitochondrial protein levels. A second strong signal in our system – NO synthesis (**Table 3** **;** **Figs. 8** **,** **9**) – provides a possible explanation for our observations. In brief, Moncada and others demonstrated in a variety of mammalian cell types that NO is a potent inducer of mitochondrial biogenesis via NO-dependent activation of guanylate cyclase, which induces PGC-1a expression [82]–[84]. Hence, strong activation of NO synthesis, which also occurs in response to Akt overexpression, could explain the early upregulation of mitochondrial proteins in myrAkt *An. stephensi*. Alternatively, direct inhibition of OXPHOS by NO results in increased AMP [67], which can activate AMP-activated protein kinase (AMPK) to increase PGC-1a expression [80]. In agreement with these results, we observed increased levels of mitochondrial proteins without increases in OXPHOS, an effect likely due to a combination of direct NO inhibition of OXPHOS and indirect RNOS-mediated damage to individual Complexes (**Table 3** **;** **Figs. 8** **,** **9**).

Conflicts in signaling – RNOS induction of mitophagy, Akt-dependent inhibition of mitophagy, Akt-dependent inhibition of mitochondrial biogenesis, NO/AMP-dependent induction of mitochondrial protein synthesis – are apparent in the midgut epithelium of myrAkt *An. stephensi*. We suggest that conflict of these signals over time results in a lack of resolution of both mitophagy and mitochondrial biogenesis, which ultimately results in energy deficits as judged by ECP. Deficits in energy would result in loss of midgut tissue architecture and electrolyte balance, both of which are critical for proper absorption of nutrients and barrier function. In support of these inferences, incomplete resolution of mitophagy is evident as shifting distributions toward increased small, round mitochondria and decreased large, round mitochondria (**Fig. 6**) from 3 d to 18 d in midguts of HM females and also in the progression of gene dosage from NTG to HT to HM females at 18 d. We suggest that these changes have resulted from fission of damaged mitochondria into small fragments that are not eliminated from the cell. Excessive fission has also been linked to *S*-nitrosylation of Drp-1 (SNO-Drp-1) [85], which could occur in the context of high level NO synthesis in myrAkt *An. stephensi*. Because proteolysis and, ultimately, mitophagy are key to resolution of oxidative damage in the cell, excessive fission and accumulated damage ultimately trigger bulk autophagy, evident as giant stalled autophagosomes in midguts from 18 d HM females (**Figs. 2** **, S1**). An imbalance of fission and fusion, whereby undamaged mitochondrial fragments are reassembled in recovered cells, has been reported in a number of human disease states. In particular, Parkinson's disease-specific proteins associated with fission/fusion include PTEN-inducible kinase 1/parkin, alpha-synuclein, and HTRA2/OMI, while mutant huntingtin appears to be associated with alterations in mitochondrial fission and fusion in Huntington's disease [86]. Based on these observations and our data, an appropriate balance of mitochondrial dynamics in the mosquito midgut epithelium – in a manner analogous to that in the gut of *C. elegans* and the midgut of *D. melanogaster* [87] – is likely to be key to overall mosquito vigor and vector capacity in malaria transmission.

Given the complexity of mitochondrial dynamics – and the conflicting signals that balance response to and recovery from damage – how can this knowledge be harnessed for novel strategies for malaria control? That is, is it possible to genetically engineer optimal mitochondrial dynamics to promote parasite killing while maintaining competitive fitness of mosquitoes under natural conditions? In light of successful manipulation of autophagy genes and mitochondrial proteins in invertebrates and mammals to alter immunity, lifespan/cell senescence, and stem cell differentiation, we suggest that this is entirely possible in vector mosquitoes. In particular, overexpression of autophagy-related genes enhances anti-pathogen immunity, including clearance of *Mycobacterium tuberculosis*-containing phagosomes in mouse macrophages and human myeloid cells *in vitro* [88], [89], protection against fatal Sindbis virus infection *in vivo* in mice ([90], and proper localization of anti-pathogen hypersensitive responses in *Arabidopsis thaliana* [91]. Hence, independent manipulation of mosquito autophagy genes is likely to impact pathogen resistance. In addition, moderate repression of neuronal ETC genes in *D. melanogaster* [92] and in neuronal and intestinal cells in *C. elegans* [93] can promote longevity, which in *C. elegans* is dependent on the upregulation of the mitochondrial unfolded protein response (mtUPR) in the nematode intestine [93]. The mtUPR is activated in response to mitochondrial stress that is communicated to the nucleus to increase the expression of the mitochondrial protein chaperones HSP-6 and HSP-60 [94]. In addition to the relationship with longevity, mtUPR-associated HSP-60 has profound effects on immunity. In particular, HSP-60 is released from damaged or stressed cells and can act a as a potent inducer of innate immune responses, including release of pro-inflammatory cytokines and NO, and is believed to be a major extracellular mediator in linking infectious agents with immune cells in response to stress [95], [96]. Further, HSP-60 peptides and protein, which bind a variety of receptors including CD36 and other class B scavenger receptors [97], have been delivered *in vivo* to enhance the immunogenicity of anti-microbial vaccines, resulting in the description of HSP-60 as a “natural adjuvant” for innate and adaptive responses to infection [98]. Intriguingly, the mosquito ortholog of HSP-60 (AGAP004002; **Table 1**), was upregulated in myrAkt *An. stephensi*, suggesting that anti-parasite resistance may be regulated in part by actions of mosquito HSP-60 via scavenger receptor binding [99], which could be enhanced directly or via ETC manipulation without epithelial damage as induced by Akt overexpression.

In this study, we used a well-defined model based on midgut-specific expression of a constitutively active myrAkt [5] to elucidate the mechanism underlying mosquito resistance to infection. Activated Akt leads to increased steady-state reactive nitrogen and oxygen species, which leads to mitochondrial dysfunction. In a compensating response, the expression of mitochondrial proteins in the midgut is upregulated, likely by PGC-1a, to promote mitochondrial biogenesis. However, this response is countered by (i) Akt-mediated repression of autophagy and (ii) the loss of ATP from mitochondria which would ensue in dysfunctional regulation of electrolyte balance and nutrient transport [57]. These two effects, which resemble “accelerated aging” [100], result in accumulation of damaged mitochondria and general loss of tissue structure and barrier function. Additionally, Akt activation inhibits apoptosis, suggesting that sustained activation of Akt would undermine elimination of damaged cells through a controlled death process [101]. Thus, a critical point for malaria resistance is quality control of mitochondrial function in the mosquito midgut, which would support – as confirmed in mammalian and invertebrate models – the maintenance of epithelial integrity through energy homeostasis, stem cell viability, and tissue repair and renewal [57], [81], [102]. Accordingly, we assert that genetic manipulation of mitochondrial processes in the midgut as a “signaling center” can be used as the basis for an efficient and novel strategy to block malaria parasite transmission.

## Materials and Methods

### Ethics statement

All protocols involving animals for mosquito rearing and feeding were approved and in accordance with regulatory guidelines and standards set by the Institutional Animal Care and Use Committee of the University of California, Davis (protocol #15990 to SL, approved on June 28, 2012) under institutional approvals by the Association for Assessment and Accreditation of Laboratory Care International (AAALAC International) accreditation program (approval #00029), the Public Health Service Office of Laboratory Animal Welfare (PHS OLAW assurance #A3433-01), and the United States Department of Agriculture Animal and Plant Health Inspection Service (USDA APHIS registration #93-R-0433). Human blood for mosquito feeding for TEM protocols was acquired as anonymously donated, expired human blood from the American Red Cross under Institutional Review Board (IRB) distribution protocol #2010-014 to MR. Anonymously donated human blood products for infection studies and midgut permeability assays were purchased from Interstate Blood Bank (Memphis, TN); these materials are deemed exempt from Human Subjects Use by the University of California Davis IRB.

### Chemicals and biochemicals

Sodium succinate, sucrose, HEPES, calcium chloride, NADH, pyruvate kinase, lactic dehydrogenase, ATP, oligomycin, rotenone, acetyl-CoA, bovine serum albumin (fatty-acid free), antimycin A, 2,3-dimethoxy-5-methyl-1,4-benzoquinone, bovine heart cytochrome c, magnesium chloride, L-arginine, 5,5′-dithiobis(2-nitrobenzoic acid), and propidium iodide were obtained from Sigma-Aldrich (St. Louis, MO). Potassium cyanide, NADPH, and oxaloacetic acid were purchased from Calbiochem/EMD (Rockland, MA). Phosphoenol pyruvate was purchased from MP Biomedicals (Solon, OH). Ferrous sulfate was purchased from Mallinckrodt (St. Louis, MO). Sodium N-methyl-D-glucamine dithiocarbamate (MGD) was purchased from the OMRF Spin Trap Source (Oklahoma City, OK). Monoclonal anti-phospho-ERK1/2 (pT183/pY185) was obtained from Sigma-Aldrich. Anti-phospho-p38 MAPK (T180/Y182) was purchased from Cayman Chemical (Ann Arbor, MI) and anti-phospho-JNK 1/2 (pT183/pY185) was purchased from Invitrogen/Life Technologies (Grand Island, NY). Anti-GAPDH antibody was purchased from Abcam (Cambridge, MA). Horseradish peroxidase-conjugated polyclonal rabbit anti-mouse IgG was purchased from Sigma-Aldrich. Horseradish peroxidase-conjugated goat anti-rabbit F(ab′)2 fragment was purchased from Invitrogen/Life Technologies (Grand Island, NY). The SuperSignal West Pico chemiluminescent detection kit was purchased from Pierce Biotechnology (Rockford, IL). All biochemical reagents were of analytical grade.

### Mosquito maintenance

Non-transgenic (NTG) as well as homozygous (HM) and heterozygous (HT) myrAkt *An. stephensi* Liston (Indian wild-type strain; myrAkt line characterized in [5]) were reared and maintained at 27°C and 75% humidity with a 16 h light and 8 h dark photoperiod. All mosquito rearing and feeding protocols were approved and in accordance with regulatory guidelines and standards set by the Institutional Animal Care and Use Committee of the University of California, Davis. For mosquito feedings, 3–5 d female mosquitoes were maintained on water or experimental treatment via soaked sterile cotton balls, changed twice daily, for 24–72 h prior to any experiment. For TEM studies mosquitoes were provided a single meal of whole human blood containing 1% sodium citrate (acquired as anonymously donated, expired blood from the American Red Cross under Institutional Review Board distribution protocol #2010-014 to MR) at 3 d after adult emergence and oviposition substrates were offered 48 h later. To obtain HT TG and NTG mosquitoes for experiments, HT male and wild-type colony female mosquitoes were mated together. The resulting progeny, consisting of approximately 50% TG and 50% NTG individuals, were reared together to eliminate differences in crowding and resources. TG and NTG siblings were separated at the pupal stage by eye fluorescence using a fluorescent dissecting microscope with DsRed filters. HM myrAkt mosquitoes were maintained as a separate line.

### MAPK western blots

For these assays, 3–5 d old NTG and HM myrAkt *An. stephensi* females were maintained on water for 24 h and then allowed to feed for 30 min on reconstituted blood provided through a Hemotek Insect Feeding System (IFS; Discovery Workshops, Accrington, UK). This blood meal contained washed human RBCs and saline (10 mM NaHCO_3_, 15 mM NaCl, pH 7.0). Midguts were dissected after blood feeding from 30 mosquitoes in each treatment group. Detection of phosphorylated MAPKs followed the protocols of Surachetpong et al. [103]. In brief, midgut protein lysates were separated by gel electrophoresis on 10% sodium dodecyl sulfate-polyacrylamide gels (SDS-PAGE), transferred to nitrocellulose membranes (BioRad, Hercules, CA), and probed for proteins of interest with target-specific antibodies. Membranes were blocked in 5% dry milk/Tris-buffered saline with 0.1% Tween-20 for 1 h at room temperature, then incubated overnight in each antibody solution. Primary and secondary antibodies, respectively, were used at the following dilutions: 1∶10,000 phospho-ERK/1∶20,000 rabbit anti-mouse IgG; 1∶1250 phospho-p38/1∶20,000 goat anti-rabbit IgG; 1∶1250 phospho-JNK/1∶20,000 goat anti-rabbit IgG; 1∶10,000 GAPDH/1∶20,000 goat anti-rabbit IgG.

### Quantitative reverse-transcriptase-PCR analyses of *Atg6*/*Atg8* mRNA

For these assays, 20–30 midguts were dissected each from non-bloodfed 18 d NTG and HM *An. stephensi*, homogenized by pulse sonication in TriZOL reagent (Invitrogen) and RNA was extracted according to the manufacturer's protocol. cDNA was synthesized from RNA samples using the SuperScript III First-Strand Synthesis System (Invitrogen) according to the manufacturer's protocol. Prior to quantitative analysis, cDNA samples were pre-amplified by PCR using gene specific primers. Cycling conditions for pre-amplification were as follows: 1× (95°C for 5 min), 20× (95°C for 30 sec, 55°C for 30 sec, 70°C for 30 sec), 1× (70°C for 5 min), 1× (4°C for 5 min). Pre-amplified cDNA was used for quantitative analysis with Maxima SYBR green/ROX qPCR Master Mix (Fermentas ThermoScientific, Waltham, MA) on an ABI 7300 real-time PCR machine. Cycling conditions for real-time PCR were as follows: 1× (95°C for 5 min), 1× (50°C for 2 min), 35× (95°C for 15 sec, 60°C for 1 min). Expression levels were calculated using the 2^−ΔΔCt^ method relative to the ribosomal protein s7 gene. Primers were designed based on published sequences from *An. gambiae* using Primer3 software: ATG6F 5′ GCGCGAGTATACGAAGCAT 3′, ATG6R 5′ GCTTCTCTAGCTGGCTCTGG 3′, ATG8F 5′ GCCATCATTCTTTGGAGAGC 3′, ATG8R 5′ TGCTATTAAAATGCGTAGAATGG 3′, RPS7F 5′ GAAGGCCTTCCAGAAGGTACAGA 3′, RPS7R 5′ CATCGGTTTGGGCAGAATG 3′.

### Preparation of NTG and HM/HT myrAkt *An. stephensi* for LC-MS/MS

A total of 50 NTG, 75 HM transgenic and 55 HT transgenic 3–5 d old adult female *An. stephensi* were each completely homogenized for total protein isolation in 1.3 ml lysis buffer (50 mM Tris HCl pH 7.5, 100 mM NaCl, 5% glycerol, 1 mM DTT, 1× SigmaFAST protease inhibitor cocktail, 1% n-octyl glucoside). Initial homogenization was achieved by 5× grinding with a PCT Shredder (Pressure Biosciences, South Easton, MA). Partially homogenized samples were placed in a Barocycler NEP2320 (Pressure Biosciences) and subjected to pressures rotating between 31,000 PSI and atmospheric for 35 cycles (20 min total). Following quantification by Bradford assay, 30 µg of total proteins from each sample were electrophoretically separated by SDS-PAGE. Three lanes (biological replicates) each were analyzed for NTG, HM and HT samples as described the Supporting Materials and Methods.

**Mitochondrial enzymatic activities** were performed on 150 hand-homogenized whole midguts from NTG and HM transgenic *An. stephensi* in a cold hypotonic solution (300 µl of 20 mM HEPES, pH 7.4, with protease inhibitors and phosphatase inhibitors). Spectrophotometry with a microplate reader (Tecan infinite M200; Tecan Systems, Inc., San Jose, CA) was used to evaluate the samples and data were recorded and analyzed with the Magellan software V6.6 (Tecan Systems, Inc.). All samples were run in triplicate on a 96-well microplate, all reagents were scaled down from 1 ml to 0.2 ml, using water or buffers as blanks, along with the modifications indicated below. Rates were expressed as nmol×(min×mosquito midgut)^−1^. Values with CV >10% were excluded from calculations and repeated with available material. Complex activities were analyzed as described in the Supporting Materials and Methods.

### Determination of adenine nucleosides and nucleotides

Extraction of ATP, ADP, AMP, NAD, and NADH were carried with two 1 ml whole mosquito midgut suspensions. One vial was spiked with 7.5 nmol of each standard during the extraction to determine their recovery. The two vials (spiked and unspiked) were extracted in parallel. The suspensions were resuspended and centrifuged at 4°C and 190×g for 3 min. Supernatant was discarded and the pellet was resuspended in 1 ml of ice-cold PBS buffer, pH 7.4, followed by a 3 min spin at 4°C and 190×g. Supernatant was discarded and the cell pellet was treated with 75 µl of ice-cold 0.5 M HClO_4_ (Sigma-Aldrich). Both vials were incubated on ice for 2 min. The suspension was centrifuged for 3 min at 4°C and 2000×g. The supernatant was kept cold and the pellet was extracted a second time by resuspending the cell pellet in another 75 µl of ice-cold 0.5 M HClO_4_, keeping on ice for 2 min, and centrifuging for 3 min at 4°C and 2000×g. The supernatants were collected and neutralized to pH 6.5 by adding ice-cold 2.5 M KOH (JT Baker/Avantor, Center Valley, PA) in 1.5 M K_2_HPO_4_ (Fisher Scientific, Fairlawn, NJ), and stored on ice for 15 min. The KClO_4_ precipitate was removed by centrifuging at 2000×g for 1 min at 4°C. The clear supernatant was filtered through a 0.45 µm nylon microspin filter (Grace, Deerfield, IL) and centrifuged for 10 min at 10,000×g at 4°C. Filtered supernatants were then spiked with 4 nmol of hypoxanthine (Sigma-Aldrich) to serve as a loading control to normalize the areas of the standards. Samples were analyzed immediately. Preparation of standards, peak identification, quantification, and HPLC conditions for these assays is described in the Supporting Materials and Methods.

### Nitrotyrosine and ATPase beta subunit western blots

These protocols were published previously [30], [31]. In brief, proteins were denatured in SDS-PAGE sample buffer (BioRad) plus 0.5% β -mercaptoethanol at 100°C for 3 min. Thirty µg of whole midgut protein were loaded onto 12% SDS-PAGE gels (BioRad) and electrophoretically separated at 200 V for approximately 50 min. Proteins were then transferred via semi-dry transfer (20% methanol, 0.0375% SDS) to a 0.45 µm PVDF membrane for 30 min at 15 V, 300 mA. Membranes were washed once for 5 min in Tris-buffered saline plus tween-20 (TBST; 150 mM NaCl, 25 mM Tris, pH 7.4, 0.1% Tween-20), blocked in 5% nonfat dry milk TBST for 1 h and then incubated with anti-nitrotyrosine (1∶1,000 dilution; EMD Millipore, Billerica, MA) or anti-beta subunit ATPase (1∶5,000 dilution; BD Biosciences) antibodies overnight at 4°C. Membranes were washed 3× for 5 min with TBST and then incubated with goat anti-mouse HRP antibody (1∶10,000; Zymed/Invitrogen Grand Island, NY) for 1 h at room temperature. After washing for 3× for 10 min with TBST proteins were then visualized with chemiluminescent reagents (ECL) on a Kodak 2000 MM Imager. The loaded protein amounts were plotted against the densitometry readings to ensure that the ECL response was within a linear range of the protein range. Images were analyzed with the Kodak Imager 2000 MM software provided by the manufacturer. All values were normalized to actin or VDAC1 as loading controls. Data were obtained from triplicates performed on different days. Statistical analyses were performed by using the Student's t-test, alpha = 0.05.

### Electron paramagnetic resonance with spin trapping technique

Whole midguts from 150 NTG and 150 HM *An. stephensi* were each hand-homogenized in 300 µl of 20 mM HEPES, pH 7.4 with protease inhibitors and phosphatase inhibitors), then incubated for 3 h at 20–22°C following addition of 100 µl reaction buffer (3 mM sodium *N*-methyl-D-glucamine dithiocarbamate [MGD] complexed with ferrous sulfate prepared fresh, 0.1 mM NADPH, 1 mM calcium chloride and 1 mM L-arginine in degassed 20 mM HEPES, pH 7.4; [104]). After the incubation, 50 to 100 µl of sample was loaded into an EPR tube and measured using a Bruker EPR and XEpr software. The conditions were as follows: average of 2 scans, sampling time of 0.163 s, sample temperature was 105 K, field modulation amplitude at 0.0008 T, field modulation of 100,000 Hz, microwave frequency of 9.4E9, microwave power of 0.0126 W, receiver gain 60, receiver time constant was 1.31 s, receiver phase of 0 deg, receiver harmonic 1, and receiver offset of 0% FS.

### Transmission electron microscopy and morphometric analysis of mitochondria

Midguts were dissected from female mosquitoes 3 d and 18 d post adult emergence from all three treatment groups (non-transgenic, HT myrAkt, and HM myrAkt) into 1× TBS buffer (0.025 M Tris, 0.15 M sodium chloride, pH 8.0). Midguts were immediately fixed in 2.5% glutaraldehyde in 0.1 M PIPES buffer pH 7.4 for 1 h at room temperature and then transferred to 0.1 M PIPES buffer, pH 7.4 on ice for same day submission to the AHSCI Imaging Core Facility at University of Arizona. Five midguts per genotype for 3 d mosquitoes and four midguts per genotype for 18 d mosquitoes were processed as follows. Samples were incubated in 1% osmium tetroxide in 0.1 M PIPES for 1 h and then washed 3 times for 5 min in deionized water. Washed samples were then transferred to 2% aqueous uranyl acetate for 20 min, rinsed in deionized water for 5 min and incubated in increasing concentrations of ethanol: 50% ethanol for 5 min, 70% ethanol for 5 min, 90% ethanol for 5 min, 100% ethanol for 5 min; 100% ethanol for 20 min, and 100% ethanol for 5 min. This was followed by three incubations (5 min each) in propylene oxide, followed by an overnight incubation in EmBed 812/propylene oxide (1∶1). The next day samples were incubated 3 times for 60 min in EmBed 812 resin, and, finally, flat embedded for 24 h at 60°C. Semi-thin sections (0.5 µm) were stained with 1% toluidine blue and examined with light microscopy. Thin silver sections were cut onto uncoated 150 mesh copper grids, stained with 2% aqueous lead citrate for 2 min and examined with a CM12S electron microscope operated at 80 kv. TIFF images were collected with an AMT 4Mpix CCD camera.

Twenty adjacent images from individual posterior midguts were acquired at 15,000× magnification. For each individual midgut, 12 to 17 adjacent images (equivalent to ∼5–8 cells) were analyzed for morphometric analysis, with care taken to ensure that areas of the analyzed midguts were the same size for each mosquito. For each micrograph all mitochondria were outlined by hand using ImageJ software [105]. The area of each individual mitochondrion, the total mitochondrial area and the total number of mitochondria were determined using ImageJ. The numbers of round (presumably damaged) and elongated (healthy) mitochondria were counted for each image, and round mitochondria were further classified by size (<50 K, 50–100 K, and >100 K nm^2^). Vacuoles with electron dense content, small stalled autophagosomes with membrane material, large stalled autophagosomes with membrane material and giant stalled autophagosomes with brush border were counted for all midguts. Two 95% confidence intervals (CI) were constructed using NTG values at 3 and 18 d ([4.4, 17.9] and [7.7, 33.8]) to identify posterior midguts that contained a number of autophagosomes above the highest 95%CI limit. Chi-square test (alpha = 0.5) was used to compare NTG versus HT, NTG versus HM, and HT versus HM. In addition, the area of each midgut epithelial cell on each micrograph, the length of the brush border, and depth from the brush border were measured. Total mitochondrial content was determined by dividing the total mitochondrial area in one midgut by the total area examined in that midgut. The data for mitochondria size, total mitochondrial area and total number of mitochondria were analyzed using two-way ANOVA followed by Tukey-Kramer HSD test. Proportions of round mitochondria were analyzed with ANOVA following arcsin transformation. Size distributions of round mitochondria among treatments were analyzed by contingency analysis followed by specific pairwise comparisons.

### *Plasmodium falciparum* strain NF54 culture and mosquito infection

Cultured parasites were grown in 10% heat-inactivated human serum and 6% washed human red blood cells (RBCs; Interstate Blood Bank, Memphis, TN) in RPMI 1640 with HEPES (Gibco) and hypoxanthine for 15 days, or until stage V gametocytes were evident. Exflagellation rates of mature gametocytes were evaluated on the day prior to and the day of mosquito infection. Mosquitoes were fed on mature gametocyte cultures diluted with human RBCs and heat-inactivated human serum (Interstate Blood Bank) for 30 min. Experimental treatments were added to the diluted culture just before blood feeding. After feeding, blood fed mosquitoes were maintained on the same treatments, provided as water-soaked sterile cotton balls and changed twice daily, until dissection. Protocols involving the culture and handling of *P. falciparum* for mosquito feeding were approved and in accordance with regulatory guidelines and standards set by the Biological Safety Administrative Advisory Committee of the University of California, Davis.

### NOS inhibition of *P. falciparum* infection in *An. stephensi*

For these studies, 125 NTG and 125 HM myrAkt *An. stephensi* were provided with water, *N* ^ω^-Nitro-*L*-arginine methyl ester (1 mg/ml, *L*-NAME; Sigma-Aldrich) or the biologically inactive *D*-NAME (1 mg/ml; Sigma-Aldrich) from 72 h before blood feeding through *P. falciparum* infection and thereafter until dissection. This experiment was repeated four times with four separate cohorts of mosquitoes. After 10 d, fully gravid females were dissected and midguts were stained with 0.1% mercurochrome to visualize *P. falciparum* oocysts. Oocysts were counted for each midgut and mean oocysts per midgut (infection intensity) and percentages of infected mosquitoes (infection prevalence; infection defined as at least one oocyst) were calculated for all dissected mosquitoes. Infection prevalence data were analyzed by Fisher's exact test to determine whether infection status differed between treatment conditions. Infection intensity data were analyzed by ANOVA to determine whether the oocysts per midgut in the controls differed among replicates. Since no differences were evident, the data were pooled across replicates. The numbers of infected mosquitoes in the water and *D*-NAME groups were outside the 95% confidence intervals for the NTG (1.79,3.05) and *L*-NAME (1.41,4.01) groups and, therefore, were excluded from analyses of the latter data. Oocyst counts between *L*-NAME and NTG groups were compared using the Mann-Whitney U test for non-parametric data.

### *L*-NAME/*D*-NAME effects on growth of *P. falciparum in vitro*

To determine whether *L*-NAME or *D*-NAME experimental treatments had a direct effect on parasite growth that could contribute to the infection phenotypes in *An. stephensi*, aliquots of *P. falciparum* NF54 culture were synchronized and subjected to a standard growth assay [103]. After synchronization, parasites were plated in 96-well flat-bottom plates in complete RPMI 1640 with HEPES, hypoxanthine, and 10% heat inactivated human serum. Parasites were treated for 48 h at 37°C with equivalent volumes of PBS and *L*-NAME or inactive *D*-NAME at 0.74 or 3.7 mM (latter concentration provided in the infectious blood meal to *An. stephensi*). Assays were terminated by replacing culture media with RPMI 1640/1% formalin. Infected RBCs were stained with 10 µg/ml of propidium iodide in phosphate-buffered saline (PBS; Cellgro, Manassas, VA) for 1 h at room temperature, then counted with FACS Calibur flow cytometer, Becton Dickinson (BD Biosciences, San Jose, CA). Relative levels of parasite growth in response to treatment were normalized to PBS-treated controls, which were set to 100%.

### Timing of malaria parasite killing in myrAkt HM *An. stephensi*

To determine the timing of parasite killing in myrAkt HM *An. stephensi* relative to NTG controls, we examined infection of HM and NTG mosquitoes with GFP-expressing *Plasmodium yoelii yoelii* 17XNL and with *P. falciparum* NF54. For *P. y. yoelii* studies, CD1 mice were infected with the 17XNL strain stably transfected with green fluorescent protein [72] and parasitemia and gametocytemia were monitored daily via Giemsa staining of thin blood films. At approximately 9 d post infection (parasitemia of 15–18%), mice were anesthetized and used to feed laboratory-reared 3–5 d, female HM and NTG *An. stephensi* (n = 125 per group). Mosquitoes were maintained on water for 24 h prior to blood feeding, and then allowed to feed for 30 min (mice were rotated among cartons to ensure uniform infections). At 6, 20 and 48 h post-feeding, midguts were dissected into PBS (Cellgro). As an experimental control, HM and NTG mosquitoes were also fed on uninfected CD1 mice. Three pools of 10 midguts were collected at each time point, homogenized using QIAshredder homogenizer columns (Qiagen, Valencia, CA), serially diluted and then scanned at 485 nm excitation/535 nm emission wavelengths. Additionally, infected and uninfected blood was collected directly from mice via cardiac puncture and fluorescence readings were obtained for serial dilutions of this blood. Parasitemia and hematocrit counts were determined by Giemsa stained thin blood smears and by hemocytometer, and the fluorescent values used as a standard for *P. yoelii* fluorescence in midgut samples. This experiment was repeated three times with three separate cohorts of mosquitoes and infected mice.

For assessment of timing for *P. falciparum* killing, laboratory-reared 3–5 d female myrAkt HM and NTG *An. stephensi* (n = 150 per group) were maintained on water for 24 h prior to blood feeding. TG and NTG mosquitoes were provided blood meals containing *P. falciparum* NF54-infected RBCs and allowed to feed for 30 min. Midguts from blood-fed females were dissected into TriZOL reagent (Invitrogen) at 6, 18 and 48 h post infection and homogenized using pulse sonication. RNA was extracted from homogenates following the manufacturer's protocol. Contaminating genomic DNA was removed from RNA samples using Turbo DNAse Free Kit (Applied Biosystems/Life Technologies, Foster City, CA). Reverse transcription was carried out with the Superscript III cDNA Synthesis Kit (Invitrogen). Real time quantitative PCR reactions were performed using Fermentas Maxima SYBR Green Master Mix (Fermentas ThermoScientific, Waltham, MA). Reactions were performed in 25 µl volumes containing 200 µg cDNA and 0.5 µM gene specific primers. Each cDNA sample was analyzed in triplicate (all Cts within 0.5 units to confirm amplification consistency) on an Applied Biosystems 7300 Real-Time PCR System. Primers were based on Berry et al. [106] to *P. falciparum* genes *Pfs16*, *Pfs25*, and *A18S* rRNA. PCR cycling conditions were as follows: 50°C/2 min; 95°C/10 min; 50 cycles with 95°C/15 sec denaturing and 60°C/1 min annealing-elongation. Since expression of *A18S* rRNA is high in all parasite stages and varies with the parasitemia [106], this gene and the ribosomal protein S7 gene (*An. stephensi*) were used to normalize parasite gene target data. Each plate also included no template negative controls. These assays were completed with four separate cohorts of *P. falciparum*-infected *An. stephensi*.

### Functional assay of midgut permeability in myrAkt HM *An. stephensi*

Laboratory reared 3–5 d old female NTG or HM mosquitoes were kept on water for 48 h and then allowed to feed for 30 min on reconstituted human blood meals [1∶1 washed human RBCs (Interstate Blood Bank) in PBS (Cellgro)] with 1×10^7^ fluorescent beads/ml (3.0–3.4 µm, Sphero Rainbow Calibration particles RCP-30-5A-2; Spherotech, Lake Forest, IL) provided through a Hemotek Insect Feeding System (Discovery Workshops). Non-blood fed mosquitoes were removed and, at 48 h post blood feeding, samples of three whole mosquitoes or three dissected midguts were placed in cell lysis buffer (Invitrogen), pulse sonicated, and filtered through a 35 µm nylon mesh to remove tissue debris. Pelleted samples were rinsed once with phosphate-buffered saline and then analyzed by flow cytometry. Data acquisition was performed with a FACScan flow cytometer (BD Biosciences), and analysis was conducted using FlowJo software (version 6.4.1; Tree Star, Ashland, OR). The number of beads per three midguts was quantified and subtracted from each analyzed sample of three whole mosquitoes to remove the contribution of beads remaining in the midgut to whole body bead counts. Statistical significance was determined by Student's t-test.

## Supporting Information

Figure S1 **Over-expression of myrAkt was associated with the appearance of stalled autophagosomes.** (A) Greater detail of a stalled autophagosome (SA) showing the composition of the SA as stacked sheets of membranous material. Image is from posterior midgut cell of 18 d old HM myrAkt *An. stephensi* taken at 15,000×. (B) Example of invagination of the midgut brush border into a giant stalled autophagosome (GSA) from a posterior midgut cell of an 18 d old HT myrAkt female. Image was captured at 2650×. Posterior midgut epithelium microvilli or brush border, BB; basal lamina mitochondria, M; stalled autophagosomes, SA; giant autophagosomes with brush border inside, GSA.
(TIF)

Figure S2 **Amino acid alignments of insect and human ATPase beta subunit (ATPB) revealed high sequence homology for cross-species detection.** Alignment of representative ATPB orthologs was performed by retrieving primary sequences from the SwissProt database and aligning them with CLUSTALW. The position of the critical tyrosine for nitration and aromatic amino acids are shown in gray highlighting. Identical positions are labeled with asterisks, whereas similar ones are indicated by one or two dots. Q17FL3 = *Aedes aegypti* ATPB; E3XEC7 = *Anopheles darlingi* ATPB; Q05825 = *Drosophila melanogaster* ATPB; P06576 = *Homo sapiens* ATPB. An ortholog from *Anopheles gambiae* was not included because the two sequences currently attributed to the ATPase alpha/beta chains for this mosquito species (Q7PKD7, Q7PZV3) are fragments and had not been reviewed (as of 8/21/2012 on SwissProt database).
(TIF)

Figure S3 ***L*** **-NAME and** ***D*** **-NAME treatment did not affect growth of asexual-stage** ***P. falciparum*** **.** Replicate cultures of *P. falciparum* NF54 were incubated for 48 h and 96 h with 0.74 mM or 3.7 mM *L*-NAME or *D*-NAME. Relative growth was compared to the water control, which is set at 100%. Data from three independent experiments were analyzed by ANOVA and by Dunnett's Multiple comparison test (alpha = 0.05) for pairwise comparisons. No significant differences among treatment groups and controls were observed.
(TIF)

Figure S4 **HPLC chromatogram of a mixture of nucleotide standards.** Standards containing 1 mM of each metabolite (ATP, ADP, AMP, NAD, NADH, and hypoxanthine) were followed at 265 nm. Retention times for these standards are shown in the table.
(TIF)

Text S1 **Supporting information.** Supplementary materials and methods.
(DOCX)
