# Supplementary figures and images for: Sustained Activation of Akt Elicits Mitochondrial Dysfunction to Block Plasmodium falciparum Infection in the Mosquito Host
Source: PLoS Pathog. 2013 Feb 28;9(2):e1003180. doi: 10.1371/journal.ppat.1003180 (PMC3585164; doi:10.1371/journal.ppat.1003180)

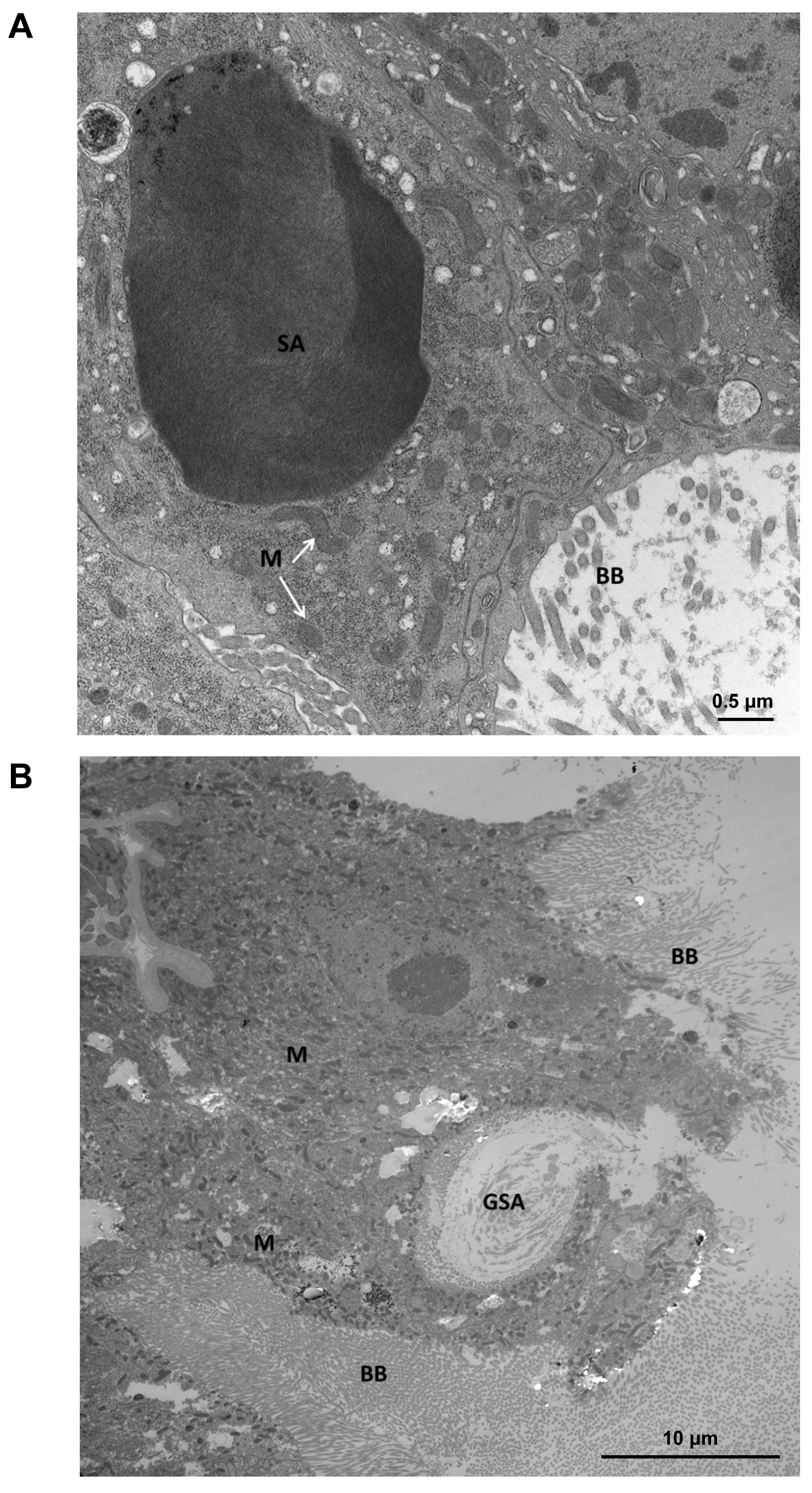

Supplement: Figure S1 — Over-expression of myrAkt was associated with the appearance of stalled autophagosomes. (A) Greater detail of a stalled autophagosome (SA) showing the composition of the SA as stacked sheets of membranous material. Image is from posterior midgut cell of 18 d old HM myrAkt An. stephensi taken at 15,000×. (B) Example of invagination of the midgut brush border into a giant stalled autophagosome (GSA) from a posterior midgut cell of an 18 d old HT myrAkt female. Image was captured at 2650×. Posterior midgut epithelium microvilli or brush border, BB; basal lamina mitochondria, M; stalled autophagosomes, SA; giant autophagosomes with brush border inside, GSA. (TIF) [file ppat.1003180.s001.tif]

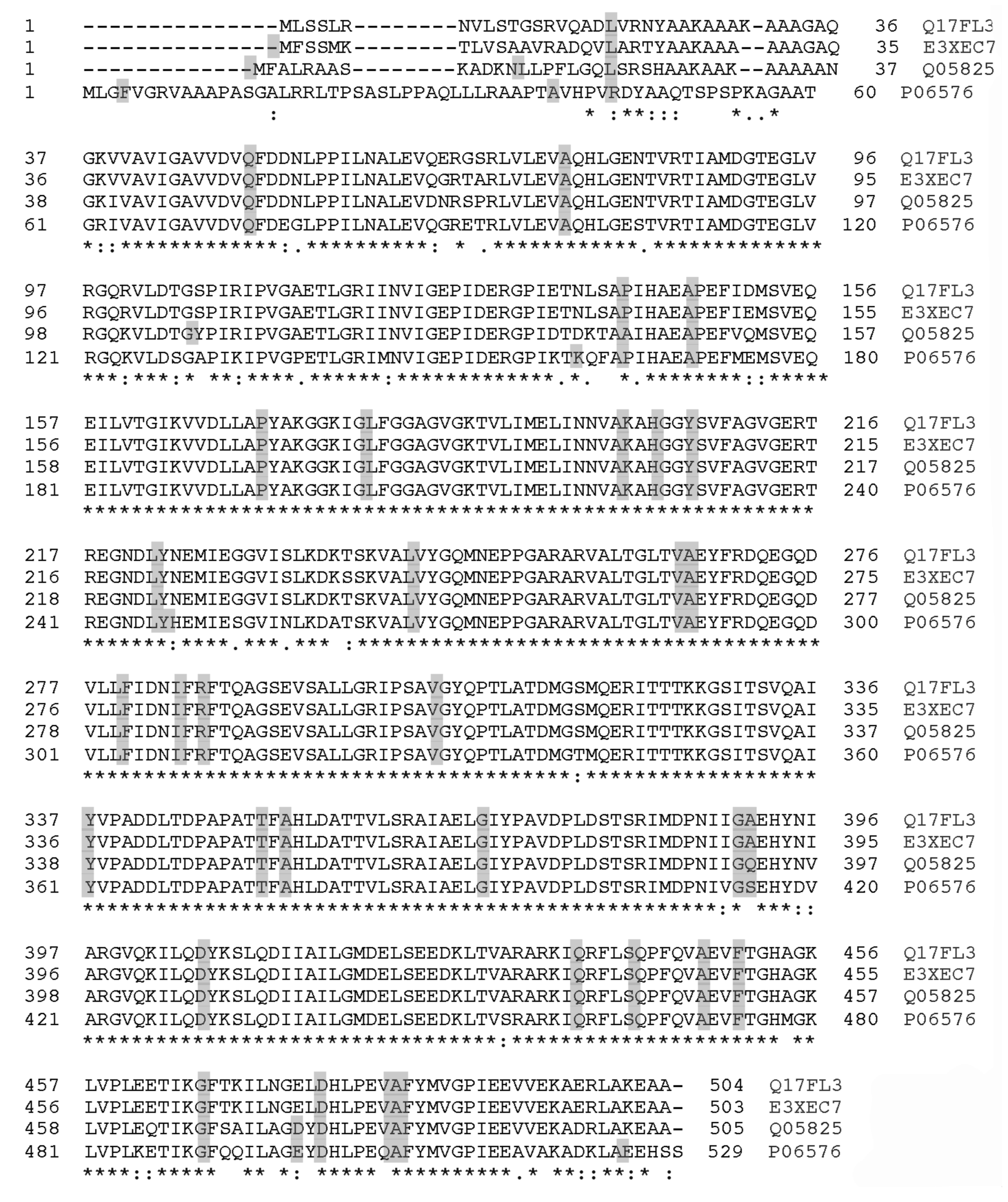

Supplement: Figure S2 — Amino acid alignments of insect and human ATPase beta subunit (ATPB) revealed high sequence homology for cross-species detection. Alignment of representative ATPB orthologs was performed by retrieving primary sequences from the SwissProt database and aligning them with CLUSTALW. The position of the critical tyrosine for nitration and aromatic amino acids are shown in gray highlighting. Identical positions are labeled with asterisks, whereas similar ones are indicated by one or two dots. Q17FL3 = Aedes aegypti ATPB; E3XEC7 = Anopheles darlingi ATPB; Q05825 = Drosophila melanogaster ATPB; P06576 = Homo sapiens ATPB. An ortholog from Anopheles gambiae was not included because the two sequences currently attributed to the ATPase alpha/beta chains for this mosquito species (Q7PKD7, Q7PZV3) are fragments and had not been reviewed (as of 8/21/2012 on SwissProt database). (TIF) [file ppat.1003180.s002.tif]

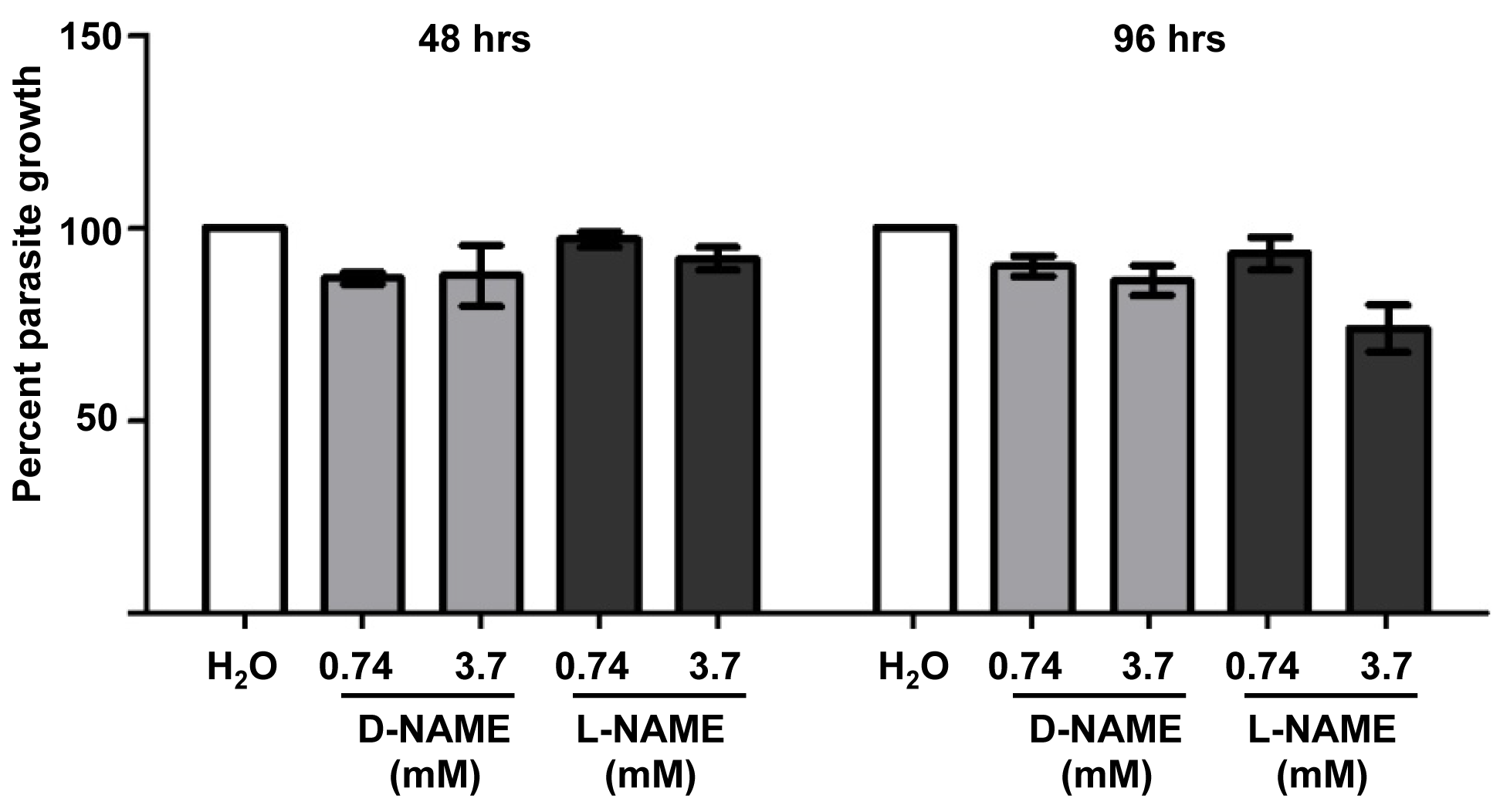

Supplement: Figure S3 — L -NAME and D -NAME treatment did not affect growth of asexual-stage P. falciparum . Replicate cultures of P. falciparum NF54 were incubated for 48 h and 96 h with 0.74 mM or 3.7 mM L-NAME or D-NAME. Relative growth was compared to the water control, which is set at 100%. Data from three independent experiments were analyzed by ANOVA and by Dunnett's Multiple comparison test (alpha = 0.05) for pairwise comparisons. No significant differences among treatment groups and controls were observed. (TIF) [file ppat.1003180.s003.tif]

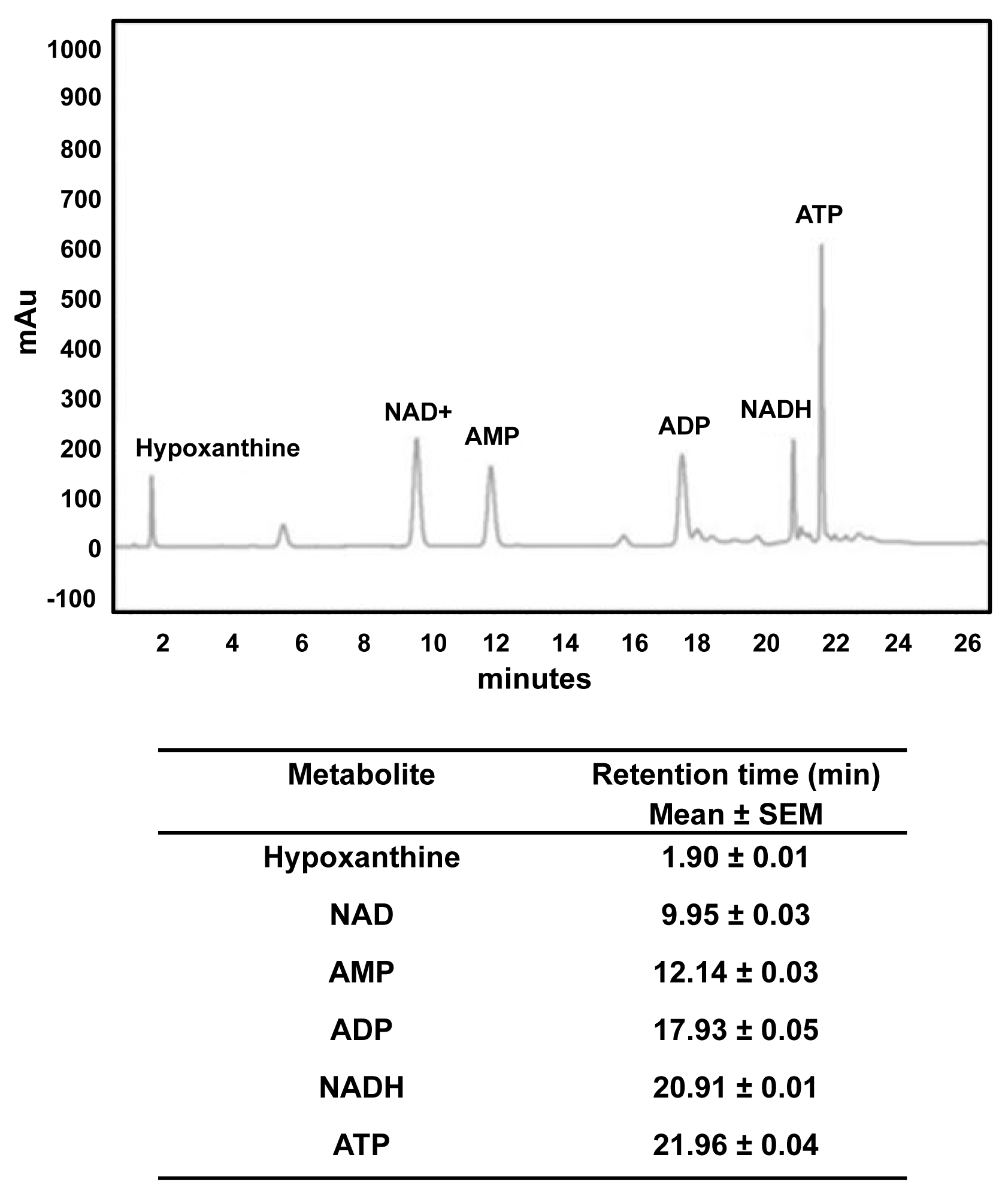

Supplement: Figure S4 — HPLC chromatogram of a mixture of nucleotide standards. Standards containing 1 mM of each metabolite (ATP, ADP, AMP, NAD, NADH, and hypoxanthine) were followed at 265 nm. Retention times for these standards are shown in the table. (TIF) [file ppat.1003180.s004.tif]
