# Supplementary material for: Sustained Activation of Akt Elicits Mitochondrial Dysfunction to Block Plasmodium falciparum Infection in the Mosquito Host
Source: PLoS Pathog. 2013 Feb 28;9(2):e1003180. doi: 10.1371/journal.ppat.1003180 (PMC3585164; doi:10.1371/journal.ppat.1003180)
Supplement: Text S1 — Supporting information. Supplementary materials and methods. (DOCX) [file ppat.1003180.s005.docx]

**Text S1: Supporting Information**

**Sustained Activation of Akt Elicits Mitochondrial Dysfunction to Block *Plasmodium falciparum* Infection in the Mosquito Host**

Shirley Luckhart^1†^*^,^ Cecilia Giulivi^2,3^*, Anna L. Drexler^1^, Yevgeniya Antonova-Koch^4^, Danielle Sakaguchi^2^, Eleonora Napoli^2^, Sarah Wong^2^, Mark S. Price^1^, Richard Eigenheer^5^, Brett S. Phinney^5^, Nazzy Pakpour^1^, Jose E. Pietri^1^, Kong Cheung^1^, Martha Georgis^1^, and Michael Riehle^4^

^1^Department of Medical Microbiology and Immunology, School of Medicine, University of California, Davis, USA 95616

^2^Department of Molecular Biosciences, School of Veterinary Medicine, University of California, Davis, USA 95616

^3^Medical Investigations of Neurodevelopmental Disorders (MIND) Institute, University of California, Davis, CA USA 95616

^4^Department of Entomology, University of Arizona, Tucson, AZ USA 85721

^5^Genome and Biomedical Sciences Center, University of California, Davis, USA 95616

*These authors contributed equally to this work.

^†^Corresponding author: Department of Medical Microbiology and Immunology

3437 Tupper Hall, One Shields Avenue, School of Medicine, University of California, Davis

Davis, CA 95616 USA, Tel: (530) 754-6963, Fax: (530) 752-8692, Email: [sluckhart@ucdavis.edu](mailto:sluckhart@ucdavis.edu)

**This file includes:**

**Supporting Materials and Methods**

**Materials and Methods**

**Preparation of NTG and HM/HT myrAkt *An. stephensi* for LC-MS/MS**

**In-gel trypsin digestion of mosquito proteins** Following electrophoresis of NTG, HM and HT *A. stephensi* proteins, gel lanes were excised into 2-3 cm sections for in-gel digestion with trypsin. Gel sections were minced into 1mm^3^ pieces and washed with 100 mM NH_4_HCO_3_. After discarding the buffer, gel pieces were dehydrated with 100% acetonitrile (ACN) for at RT for 15 min. Sample gel pieces were dried completely in a rotary evaporator prior to rehydration in 100mM NH_4_HCO_3_ with 10mM DTT at 56°C for 30 minutes to reduce the sample. The DTT solution was decanted and the sample gel pieces were rehydrated again in acetonitrile and dried completely. Sample gel pieces were alkylated on cysteine residues using 55 mM iodoacetamide in 100mM NH_4_HCO_3_ (20 min in the dark, RT). After alkylation the supernatant was discarded and the sample gel pieces were washed twice briefly with 100mM NH_4_HCO_3_. The supernatant was decanted and 100% acetonitrile was added to the sample gel pieces for 15 min at RT. Following complete drying, the sample gel pieces were rehydrated in digestion buffer of 50 mM NH_4_HCO_3_ containing 13 ng/μl sequencing grade modified trypsin (Promega) and incubated overnight at 37°C. Digested sample gel pieces were centrifuged briefly to collect the supernatants which were transferred into Protein Lo-Bind tubes (Eppendorf). The remaining sample gel pieces were immersed in 60% ACN/1% TFA and then sonicated in Branson 1200 ultrasonic water bath for 10 min. Supernatants from sonicated gel pieces were collected and added to the primary supernatants in Lo-Bind tubes. Peptides were dried in a vacuum concentrator after digestion, and then resolubilized in 2% acetonitrile/0.1% trifluoroacetic acid for LC-MS/MS analysis, using a 10-min sonication in the Branson 1200.

**LC-MS/MS** Digested peptides were analyzed by LC-MS/MS on a Thermo Finnigan LTQ with Michrom Paradigm LC and CTC Pal autosampler. Peptides were separated using a Michrom 200 µm x 150 mm Magic C18AQ reversed phase column at 2 µl/min. Peptides were directly loaded onto an Agilent ZORBAX 300SB C18 reversed phase trap cartridge, which, after loading, was switched in-line with a Michrom Magic C18 AQ 200 μm x 150 mm column connected to a Thermo-Finnigan LTQ iontrap mass spectrometer through a Michrom Advance Plug and Play nano-spray source. The nano-LC column was run with a 45 min-long gradient using a two buffer system, with Buffer A being 0.1% formic acid and Buffer B 100% ACN. The gradient was 2-35% buffer B in 30 min, 35-80% buffer B in 2 min, 80% buffer B for 1 min, 80-2% buffer B in 0.5 min, and 2% buffer B for 11.5 min at a flow rate of 2 ml/min for the maximum separation of tryptic peptides. MS and MS/MS spectra were acquired using a top 10 method, where the top 10 ions in the MS scan were subjected to automated low energy CID. An MS survey scan was obtained for the m/z range 375-1300. An isolation mass window of 2 Da was for the precursor ion selection, and a normalized collision energy of 35% was used for the fragmentation. A 2 min duration was used for the dynamic exclusion.

**Protein identification validation and differential expression** Tandem mass spectra were extracted, charge state deconvoluted and deisotoped by Mass Matrix version 3.9. All MS/MS samples were analyzed using X! Tandem (The GPM, thegpm.org; version CYCLONE [2011.05.01.1]). X! Tandem was set up to search the Uniprot *An. gambiae* reference proteome set (May 2012; 13,072 entries), the *An. stephensi* preliminary data set ([www.vectorbase.org](http://www.vectorbase.org); June 2012) and the cRAP database of common laboratory contaminants ([www.thegpm.org/crap](http://www.thegpm.org/crap); 114 entries) plus an equal number of reverse protein sequences assuming the digestion enzyme trypsin. X! Tandem was searched with a fragment ion mass tolerance of 0.40 Da and a parent ion tolerance of 1.8 Da. Iodoacetamide derivative of cysteine was specified in X! Tandem as a fixed modification. Deamidation of asparagine and glutamine, oxidation of methionine and tryptophan, sulphone of methionine and tryptophan oxidation to formylkynurenin of tryptophan were specified in X! Tandem as variable modifications. The point mutation option in x! Tandem was turned on.

**Criteria for protein identification** Scaffold (version Scaffold_3.5.1, Proteome Software Inc., Portland, OR) was used to validate MS/MS based peptide and protein identifications. Peptide identifications were accepted if they could be established at greater than 80.0% probability as specified by the Peptide Prophet algorithm [1]. Protein identifications were accepted if they could be established at greater than 95.0% probability and contained at least 2 identified peptides. Protein probabilities were assigned by the Protein Prophet algorithm [2]. Proteins that contained similar peptides and could not be differentiated based on MS/MS analysis alone were grouped to satisfy the principles of parsimony. Using the parameters above, the False Discovery Rate (FDR) was calculated to be 5.4% on the protein level and 0.2% on the peptide level for the *An. gambiae* search set and 5.7% on the protein level and 0.3% on the peptide level for the *An. stephensi* search set [3].

**Differential protein expression using label free spectral counting** Differential protein expression among NTG, HM and HT were calculated using the ANOVA feature of the Scaffold program based on the total number of assigned spectra per protein.

**Mitochondrial enzymatic activities**

**Complex activities** Complex I activity was evaluated either by measuring the NADH-decylubiquinone oxidoreductase (NQR) activity or the NADH ferricyanide reductase (NFR) activity as described [102]. For NQR activity measurements, 10 to 15 µg of protein were added to 150 µl of water and incubated for 2 min at 37°C prior to adding 40 µl of reaction buffer consisting of 5 mg/ml BSA, 240 µM KCN, 4 μM antimycin A, 50 mM HEPES, pH 7.5, 50 µM 2,3-dimethoxy-5-methyl-1,4-benzoquinone (CoQ_0_), and either 4 µM rotenone dissolved in ethanol (rotenone) or an equal volume of ethanol without rotenone (vehicle). The reaction was started by adding 0.15 mM NADH. The assay was measured at 340 nm following the oxidation of NADH at 37°C for 10 min. Rotenone-sensitive activities were calculated from the linear part of ∆A versus time plots and using an extinction coefficient of 6.22 mM^-1^ cm^-1^. The linear slope value for the activity without rotenone (vehicle) was subtracted from the value with rotenone. NFR activity was evaluated by following the reduction of ferricyanide at 412 nm. Ten to 15 µg were added to each well of a 96-well microplate in the presence of 190 μl of reaction buffer consisting of 2.5 mg/ml BSA, 3.6 μM antimycin A, varying concentrations of potassium ferricyanide (0.25 mM, 0.5 mM, 0.75 mM, and 1.0 mM), 2 mM KCN, 5 mM MgCl_2_, 20 mM HEPES, pH 7.5. The reaction was started with the addition of 0.1 mM NADH and followed for 5 min. The measurements were followed at 37°C in an Infinite 200 microplate reader (Tecan Systems, Inc.) for 10 min. Activity was calculated using the y-intercept from a linear fit of delta absorbance vs. concentration of potassium as 1/V_max_, using an extinction coefficient of 1000 M^-1^ cm^-1^.

Succinate-cytochrome c reductase (SCCR) activity was used to evaluate Complex II-III by following the reduction of 2,6-dichlorophenolindophenol at 600 nm and 30°C. Ten to 15 µg of mosquito midgut protein was added to 10 mM succinate, 240 µM KCN, 4 µM rotenone and either 1 mM 2-thenyltrifluoroacetone (TTFA) in ethanol or an equal volume of ethanol without TTFA, and incubated at 30°C for 10 minutes. The reaction was then initiated by the addition of 0.4 mM cytochrome *c*. The rate sensitive to TTFA was taken as Complex II activity. This rate was calculated from the linear part of ∆A versus time plots and using an extinction coefficient of 21, 000 M^-1^ cm^-1^. The linear slope value for the activity without TTFA was subtracted from the value with TTFA.

Complex V was evaluated by following ATPase activity. The assay was performed at 340 nm and 37°C following the reduction of NADH. Each well contained 10-15 µg of protein, 140 µl of reaction buffer (2.5 mM phophoenol pyruvate, 0.4 mM NADH, 2.5 mM MgCl_2_, 45 mM HEPES, pH 7.5 with 6.3 units/ml pyruvate kinase and 4.5 units/ml lactic dehydrogenase). The reaction was started with the addition of 2.5 mM ATP and followed for 10 min, in the presence either 3 μM oligomycin dissolved in ethanol (5 mg/ml) or an equal volume of ethanol without oligomycin. The rate sensitive to oligomycin was taken as Complex V activity. This rate was calculated from the linear part of ∆A versus time plots and using an extinction coefficient of 6.22 mM^-1^ cm^-1^. The linear slope value for the activity without oligomycin was subtracted from the value with oligomycin.

The activity of citrate synthase in whole mosquito midguts was evaluated exactly as described [4]. Protein concentration was evaluated for each sample using the Pierce BCA protein assay kit according to the manufacturer’s instruction.

**Determination of adenine nucleosides and nucleotides**

**Preparation of standards, peak identification and quantification** To identify and quantify peaks during analysis, 7 different concentrations of standards (0.05-1 nmol ADP, AMP, and NADH; 0.5-10 nmol NAD and ATP) were prepared containing ATP, ADP, AMP, NAD, and NADH. Standards were diluted in Mobile phase A (see below) spiked with 4 nmol of hypoxanthine (Sigma-Aldrich). Standards and samples were followed with a diode array detector at 250 and 280 nm. Peaks were identified by comparison with retention times of standards. Area of peaks for each metabolite was normalized to the area of hypoxanthine. Amount of nucleotide present per cell was calculated from the linear part of the area of nucleotide or nucleoside/area of hypoxanthine versus nmol of nucleotide or nucleoside plots and expressed as nmol/midgut. The amount of nucleotides recovered was determined to be 80 to 90% from the vials spiked with 7.5 nmol of standards. The amount of metabolites present per cell was normalized to the recovery value.

**HPLC conditions** Chromatographic conditions were taken from [Ryll](#_ENREF_2) and Wagner [5] with the modifications as below. All samples were measured in a chromatographic system consisting of two HPLC pumps (Model LC-10ADVP; Shimadzu, Columbia, MD), diode array detector (Model SPD-10AVP, Shimadzu), Auto Injector (Model SIL-10AD, Shimadzu), and a C8 column (Symmetry C8 column end-capped, 4.6 mm x 150 mm, 5 µm particle size and 100 A pore size; Waters, Milford, MA). Mobile phase A consisted of 100 mM KH_2_PO_4_-K_2_HPO_4_ buffer, pH 6.0, supplemented with 8 mM tetrabutylammonium hydrogen sulphate (Sigma-Aldrich). Mobile phase B consisted of 70% buffer A and 30% methanol (HPLC-Grade, Fisher Scientific). The gradient conditions used for the HPLC separation were as follows: 100% A for 2.5 min, 0-40% B for 14 min, 40-100% B for 1 min, 100% B for 6 min, 100-0% B for 1 min, followed by an equilibrium phase of 100% A for 8 min at a flow-rate of 1.5 ml/min. An example of a chromatogram and retention times are shown in **Fig. S4**.

**Supporting References**

1. Keller A, Nesvizhskii AI, Kolker E, Aebersold R (2002) Empirical statistical model to estimate the accuracy of peptide identifications made by MS/MS and database search. Anal Chem 74: 5383-5392.

2. Nesvizhskii AI, Keller A, Kolker E, Aebersold R (2003) A statistical model for identifying proteins by tandem mass spectrometry. Anal Chem 75: 4646-4658.

3. Tabb DL (2008) What's driving false discovery rates? J Proteome Res 7: 45-46.

4. Giulivi C, Zhang YF, Omanska-Klusek A, Ross-Inta C, Wong S, et al. (2010) Mitochondrial dysfunction in autism. JAMA 304: 2389-2396.

5. Ryll T, Wagner R (1991) Improved ion-pair high-performance liquid chromatographic method for the quantification of a wide variety of nucleotides and sugar-nucleotides in animal cells. J Chromatogr 570: 77-88.
